# Characterization of Epigallocatechin-Gallate-Grafted Chitosan Nanoparticles and Evaluation of Their Antibacterial and Antioxidant Potential

**DOI:** 10.3390/polym13091375

**Published:** 2021-04-23

**Authors:** María J. Moreno-Vásquez, Maribel Plascencia-Jatomea, Saúl Sánchez-Valdes, Judith C. Tanori-Córdova, Francisco J. Castillo-Yañez, Idania E. Quintero-Reyes, Abril Z. Graciano-Verdugo

**Affiliations:** 1 Departamento de Ciencias Químico Biológicas, Universidad de Sonora, 83000 Hermosillo, Sonora, Mexico; mariadejesus.moreno@unison.mx (M.J.M.-V.); jcastillo@guayacan.uson.mx (F.J.C.-Y.); 2 Departamento de Investigación y Posgrado en Alimentos, Universidad de Sonora, 83000 Hermosillo, Sonora, Mexico; 3 Departamento de Procesos de Transformación de Plásticos, Centro de Investigación en Química Aplicada, 25294 Saltillo, Coahuila, Mexico; saul.sanchez@ciqa.edu.mx; 4 Departamento de Investigación en Polímeros y Materiales, Universidad de Sonora, 83000 Hermosillo, Sonora, Mexico; jtanori@unison.mx; 5 Departamento de Ciencias de la Salud, Universidad de Sonora, 85040 Cd. Obregón, Sonora, Mexico; idania.quintero@unison.mx

**Keywords:** nanoparticles, modified chitosan, grafted chitosan, epigallocatechin gallate, antibacterial, antioxidant

## Abstract

Nanoparticles based on chitosan modified with epigallocatechin gallate (EGCG) were synthetized by nanoprecipitation (EGCG-g-chitosan-P). Chitosan was modified by free-radical-induced grafting, which was verified by Fourier transform infrared (FTIR). Furthermore, the morphology, particle size, polydispersity index, and zeta potential of the nanoparticles were investigated. The grafting degree of EGCG, reactive oxygen species (ROS) production, antibacterial and antioxidant activities of EGCG-g-chitosan-P were evaluated and compared with those of pure EGCG and chitosan nanoparticles (Chitosan-P). FTIR results confirmed the modification of the chitosan with EGCG. The EGCG-g-chitosan-P showed spherical shapes and smoother surfaces than those of Chitosan-P. EGCG content of the grafted chitosan nanoparticles was 330 μg/g. Minimal inhibitory concentration (MIC) of EGCG-g-chitosan-P (15.6 μg/mL) was lower than Chitosan-P (31.2 μg/mL) and EGCG (500 μg/mL) against Pseudomonas fluorescens (*p* < 0.05). Additionally, EGCG-g-chitosan-P and Chitosan-P presented higher Staphylococcus aureus growth inhibition (100%) than EGCG at the lowest concentration tested. The nanoparticles produced an increase of ROS (*p* < 0.05) in both bacterial species assayed. Furthermore, EGCG-g-chitosan-P exhibited higher antioxidant activity than that of Chitosan-P (*p* < 0.05) in 2,2′-azino-bis (3-ethyl-benzothiazoline-6-sulfonic acid) (ABTS), 2,2-diphenyl-1-picrylhydrazyl radical (DPPH) and ferric-reducing antioxidant power assays. Based on the above results, EGCG-g-chitosan-P shows the potential for food packaging and biomedical applications.

## 1. Introduction

Food loss and waste are some of the main problems facing the food industry. According to the Food and Agriculture Organization of the United Nations (FAO), one-third of food produced globally for human consumption is lost or wasted per year (approximately 1.3 billion tons) [1]. The main factor contributing to this problem is deterioration because of microbiological, enzymatic, and physicochemical reactions [2]. The deterioration process leads to changes in pH, formation of toxic compounds, gas production, slime formation, off-flavor production, and lipid oxidation. However, there are several preservation methods for increasing the shelf life of foods, such as the incorporation of synthetic active compounds, mainly synthetic antimicrobials and antioxidants. Nevertheless, there remains a strong consumer demand for natural safer and higher-quality foods. Furthermore, the increasing occurrence of new food-borne disease outbreaks has raised concerns about food safety. These concerns have led to numerous studies on the isolation, characterization, and evaluation of biologically active compounds from natural sources, such as animals, microorganisms, herbs, and plants [3].

EGCG is a biologically active compound in green tea (*Camellia sinensis*), which is categorized as “generally recognized as safe” (GRAS) by the Food and Drug Administration (FDA). It is widely considered as a potential alternative to antimicrobial and antioxidant synthetic food additives. EGCG has been shown to inhibit the growth of *Escherichia coli* [4], *Pseudomonas aeruginosa* [5], *Streptococcus mutans* [6], *Staphylococcus aureus* [7], *Vibrio cholerae* [8], *Helicobacter pylori* [9], and *Clostridium perfringens* [10]. Furthermore, EGCG is capable of inhibiting *Pseudomonas* sp. growth, which is responsible for the microbial spoilage of fresh foods, mainly meat and fish products. In addition, *Pseudomonas* is one of the least sensitive bacteria to the effect of bioactive agents from natural sources, such as spice and herbs [3]. The antioxidant activity of EGCG in vitro and in biological systems has been reported and indicated that EGCG exhibits higher antioxidant activity than synthetic(tert-butylhydroquinone (THBQ), butylated hydroxytoluene (BHT), and butylated hydroxyanisole (BHA)) and natural (carvacrol, eugenol, and thymol) antioxidants [11,12]. However, several studies have demonstrated that the biological activities of polyphenols such as EGCG depend on several factors, including pH, temperature, and UV light [4,13,14]. Nanotechnology can improve the biological properties and potential application areas of active compounds grafted to polymers, allowing them to inhibit bacterial growth and stabilize radicals at lower concentrations than the bulk material due to the increase in surface area/volume ratio [15]. By forming nanoparticles, bioactive compounds with antioxidant activity have been incorporated in a polymer matrix to protect them from degradation. For example, solid lipid nanoparticles with limonene, composed of glycerol monostearate and poloxamer 188, ameliorated both lipid peroxidation and cytotoxicity in a HaCaT cell line [16]. In a similar study, Souto et al. [17] reported that perillaldehyde’s cytotoxic effect 1,2-epoxide against MCF-7 cell line could be improved when surface-modifying the cationic lipid nanoparticles (made of compritol ATO 888, poloxamer 188, and cetyltrimethylammonium bromide) with streptavidin. These results demonstrate the nutraceutical and pharmaceutical applications of antioxidants-loaded nanoparticles.

Covalently grafting polyphenols in nanomaterial is an alternative method, which has been shown to improve the biological activities of chitosan and active compounds and enhance the stability of phenolic compounds, thus increasing the area of potential applications of chitosan [15,16,17,18]. Unlike the nanoencapsulation methods of active compounds loaded in chitosan, which involve physical interactions or weak chemical interactions, the chemical modification of chitosan with compounds with biological activity improves its solubility in water [19] by that would expand its application in nutraceuticals and pharmaceuticals. Chitosan is a biopolymer that possesses in its chemical structure reactive functional groups that are susceptible to chemical modification by covalent grafting of antioxidant/antimicrobial compounds [20,21,22]. Accordingly, the majority of related studies have reported an increase in the antimicrobial or antioxidant effects of compounds grafted onto chitosan. In our previous study [22], we showed an increase in the antibacterial activity of EGCG-grafted-chitosan compared to those of pure EGCG and chitosan alone against *S. aureus* and *Pseudomonas* sp. Furthermore, it showed higher antioxidant activity than that of chitosan. Considering that it is desirable to decrease the dose of chitosan required to obtain adequate activity, in this study, we focus on increasing the properties of the modified chitosan through the elaboration of nanoparticles by nanoprecipitation.

Nanoprecipitation is simple, fast, requires a small volume of raw material, and needs a low amount of energy for obtaining particles at nanometric and micrometric scales [23]. Nanoprecipitation represents numerous advantages compared with methods such as emulsion diffusion methods, emulsion–evaporation, and precipitation by salting-out which need a precursor emulsion and higher electric power [24]. Thus, nanoparticles prepared in this way show promises for application in the food, agricultural, and pharmaceutical industries.

Chitosan nanoparticles have employed to encapsulate active compounds such as propolis [25], EGCG [26], quercetin [27], thyme [28], caffeic acid [29], chlorogenic acid [29], rosmarinic acid [29], among others, as well as for the immobilization of enzymes [30,31]. Moreover, the synthesis of chitosan conjugate nanoparticles has been reported. However, organic solvents are required, and the graft yield is poor [15,32]. Several studies have evaluated the antioxidant and antibacterial activities of nanoparticles based on polymers covalently grafted to antioxidant/antibacterial compounds with potential application in the food industry, nevertheless as far as we know, little information regarding EGCG-conjugated chitosan-based nanoparticles prepared by the free radical grafting procedure, which is solvent-free, is observed in the literature. Accordingly, this work aims to increase the antibacterial and antioxidant activity of EGCG covalently grafted to chitosan through the elaboration of nanoparticles by nanoprecipitation. In addition, the characterization of the nanoparticles obtained and the possible mechanism of action of antibacterial activity by the production of reactive oxygen species (ROS) were evaluated.

## 2. Materials and Methods

### 2.1. Materials

Epigallocatechin gallate ((−)-Epigallocatechin-3-Gallate) with a purity of 94% *w*/*w* was supplied by Teavigo^®^, DSM nutritional Products (Kaiseraugst, Switzerland). Chitosan with average molecular weight 121 kDa, and a degree of deacetylation 80%, hydrogen peroxide (H_2_O_2_), ascorbic acid, acetic acid, 2,2-diphenyl-1-picrylhydrazyl radical (DPPH), 2,2′-azino-bis (3-ethyl-benzothiazoline-6-sulfonic acid) (ABTS), 2,4,6-Tris(2-pyridyl)-s-triazine (TPTZ), 6-hydroxy-2,5,7,8-tetramethylchroman-2-carboxylic acid (Trolox), Folin-Ciocalteu reagent, sodium carbonate and methanol were purchased from Sigma-Aldrich (Sigma Chemical Co., Saint Louis, MO, USA).

### 2.2. Bacteria Strains

The antibacterial activity was investigated against Gram-negative (*Pseudomonas fluorescens* ATCC 13525) and Gram-positive strains (*Staphylococcus aureus* ATCC 25923). The bacterial strains were obtained from the ATCC (Manassas, VA, USA). *Staphylococcus aureus* is a typical foodborne and *Pseudomonas fluorescens* is recognized as major food spoilage microorganism.

### 2.3. Chemical Modification of Chitosan

The EGCG-grafted-chitosan was synthetized based on the chemical modification of chitosan, by employing ascorbic acid/hydrogen peroxide as pair initiator system according to our previous study [22]. A 1:1 weight ratio of chitosan to EGCG was used. In a 25 mL glass flask, Chitosan was dissolved in 0.4% (*v*/*v*) aqueous acetic acid solution by stirring at 25 °C for 24 h. Then, redox pair as initiator system was added as follows: 1 mL of hydrogen peroxide (1.0 M) containing ascorbic acid (0.054 g), the mixture was maintained at 25 °C under atmospheric conditions and, after 30 min, EGCG was incorporated into the glass flask. The reaction was performed under magnetic stirring for 24 h at 25 °C. The EGCG-grafted-chitosan obtained was purified into centrifugal filter tubes (MWCO 100,000 Da) (Merck KGaA, Darmstadt, Germany) by cycles of centrifugation at 4 °C/60 min/3500 rpm (Sorvall Legend XTR Centrifuge, Eppendorf, Hamburg, Germany). The purification of EGCG-grafted-chitosan was verified by spectrophotometric analysis (254 nm) after the cycles of centrifugation. The pure EGCG-grafted-chitosan solution was frozen and then lyophilized. As a control, blank chitosan was prepared under the same conditions.

### 2.4. Synthesis of Modified and Unmodified Chitosan Nanoparticles

Nanoparticles of EGCG-grafted-chitosan and chitosan (control) were synthesized by nanoprecipitation according to Luque-Alcaraz et al. [33]. Chitosan was dissolved (0.5 mg/mL) in an aqueous phase containing 0.4% acetic acid. The aqueous phase (2.5 mL) was then added to the organic phase (40 mL methanol) by means of a needle positioned five centimeters above the surface using a peristaltic pump at 0.90 mL/min under moderate magnetic stirring. The methanol was then removed using a rotary evaporator. The nanoparticles were recovered and lyophilized prior to further characterization.

### 2.5. FTIR Analysis

FTIR analysis was performed to verify that the particle synthesis method does not affect the covalent bound between EGCG and chitosan. The EGCG-g-chitosan-P and Chitosan-P were blended with KBr and compressed to form disks. Then, the samples were analyzed using a Perkin-Elmer Spectrum 2000 spectrometer (Perkin-Elmer Co., Norwalk, CT, USA). The samples were scanned 16 times from 4000–400 cm^−1^, with a resolution of 4 cm^−1^.

### 2.6. Morphological Characterization of Nanoparticles

The morphological characterization of EGCG-g-chitosan-P and Chitosan-P was carried out by a JEOL 2010F transmission electron microscope (JEM 2010F, JEOL, Tokyo, Japan) [34].

### 2.7. Particle Size and Polydispersity Index

The particle size and polydispersity index of the EGCG-g-chitosan-P and Chitosan-P (control) were determined by dynamic light scattering (DLS) using a Zetasizer nano SERIES (ZEN 3600, Malvern Instrument Ltd., Malvern, UK), according to Luque-Alcaraz et al. [33]. The concentration of the nanoparticles was 100 μg/mL in Millipore water and the samples were sonicated for 30 s. The dynamic particle sizes were measured at 25 °C using a detection angle of 90°.

### 2.8. Zeta Potential (ζ)

The zeta potentials of the EGCG-g-chitosan-P and Chitosan-P (control) were measured on a Zetasizer nano SERIES (ZEN 3600, Malvern Instrument Ltd., Malvern, UK) on basis of the DLS technique. The samples were suspended in Milli-Q water and all measurements were performed in triplicate at 25 °C.

### 2.9. EGCG Quantification by the Folin—Ciocalteu Method

The EGCG concentration in EGCG-g-chitosan-P was determined according to Moreno-Vasquez et al. [22]. Additionally, this method was used to quantify the EGCG concentration of the solutions of EGCG-g-chitosan-P in the bacterial growth inhibition and antioxidant activity tests. The results were calculated according to Equation (1) which was obtained from a calibration curve prepared with pure EGCG solutions (0–20 μg/mL):

*y* = 0.0036*x* − 0.0251 (1)

where “*x*” is the sample’s absorption value at 760 nm and “*y*” is the concentration of grafted EGCG (μg of EGCG/g of nanoparticles).

### 2.10. Determination of Antibacterial Activity In Vitro

#### 2.10.1. Nanoparticles Stock Solutions

In the bacterial growth inhibition tests the EGCG-g-chitosan-P and Chitosan-P (control) were dispersed in Milli-Q water (10 mg/mL) and serial diluted to obtain solutions with concentrations of 10,000, 8000, 4000, 2000, 1000, 500, 250, 125, 62.5, 31.2, 15.6, 7.8, 3.9, 1.9, and 0.9 μg/mL [35]. Additionally, solutions of EGCG alone were employed as a control, and they were prepared to provide the same EGCG concentrations as those of the EGCG-g-chitosan-P solutions. The EGCG concentrations in the EGCG-g-chitosan-P solutions were 628.6, 438.3, 349.3, 287.2, 229.6, 114.8, 57.4, 28.7, 14.3, 7.1, 3.5, 1.7, 0.9, 0.4, and 0.2 μg/mL. Additionally, for MIC and MBC analysis, pure EGCG, EGCG-g-chitosan-P and Chitosan-P (control) were dispersed in Milli-Q water (10 mg/mL) and serial diluted to obtain solutions with concentrations from 10,000 to 0.9 μg/mL.

#### 2.10.2. Bacteria and Culture Conditions

The bacterial strains used in this study were *Staphylococcus aureus* ATCC 25923 (representative Gram-positive strain) and *Pseudomonas fluorescens* ATCC 13525 (representative Gram-negative strain). The bacteria were dispersed in Mueller-Hinton broth (pH 7.2). Optical density was measured using a double-beam spectrophotometer at 600 nm. The absorbance value must equal the standard absorbance value of McFarland 0.5 (10^8^ CFU/mL) and diluted with Mueller-Hinton broth to reach a final bacteria cell concentration of 10^6^ CFU/mL.

#### 2.10.3. Bacterial Growth Inhibition (%)

The effect of pure EGCG and the nanoparticles prepared from chitosan and EGCG-grafted-chitosan against the bacterial growth inhibition was evaluated according to Aljawish et al. [35] with a slight modification. The 96-well microplates were charged with the bacterial cells (210 μL) at concentration of 106 CFU/mL in Mueller-Hinton broth (pH 7.2) and pure EGCG, Chitosan-P, or EGCG-g-chitosan-P (90 μL) (pH of 5.4) was incorporate, whereupon the pH changed to 6.2. The experiment was performed in triplicate. As a control, 90 μL of sterile distilled water was used. The microplates were incubated at 37 °C for 24 h. The bacterial growth inhibition was evaluated by spectrophotometric analysis at 630 nm (Veloskan™ LUX, Thermo Fisher Scientific, Waltham, MA, USA) and calculated as inhibition percentage (%) according to Cueva et al. [36], using Equation (2):

(2) $$Bacterial\;growth\;inhibition\;\left(\%\right)=1-\frac{\left(T_{2sample}-T_{1sample}\right)-\left(T_{2blank\;}-T_{1blank}\right)}{\left(T_{2growth\;}-T_{1growth}\right)-\left(T_{2blank\;}-T_{1blank}\right)}\times 100$$

where *T*_1*sample*_ and *T*_2*sample*_ are the optical densities at 630 nm of the bacteria growth in the presence of samples before (*T*_1_) and after (*T*_2_) incubation, respectively; *T*_1*blank*_ and *T*_2*blank*_ correspond to the optical densities of Mueller-Hinton broth with samples before and after incubation, respectively; and *T*_1*growth*_ and *T*_2*growth*_ correspond to the bacteria cells in the presence of Mueller-Hinton broth (positive control) before and after incubation, respectively.

#### 2.10.4. Minimal Inhibitory Concentration (MIC) and Minimal Bactericidal Concentration (MBC)

The MIC and MBC values for EGCG and the nanoparticles (chitosan and EGCG-grafted-chitosan) were determinate according to Moreno-Vasquez et al. [22]. MICs were tested using 96-well microplates. The EGCG and nanoparticle (90 μL) solutions were incorporated into MHB medium inoculated with bacteria at 10^6^ CFU/mL (210 μL). Then, the microplates were incubated at 37 °C/24 h. MIC and MBC values were determinate using a microplate reader (Veloskan™ LUX, Thermo Fisher Scientific, Waltham, MA, USA). Differences in absorbance (630 nm) between (*T*_2*sampl*_ *− T*_0*sample*_) and (*T*_2*blank*_ − *T*_1*blank*_) lower than 0.02 were taken to indicate no bacterial growth. The MBC assay was carried out by counting the number of colonies after the bacteria were cultured for 24 h at 37 °C on Mueller-Hinton agar plates.

#### 2.10.5. Bacterial Morphometry

An aliquot from bacterial growth inhibition evaluation microplate was taken to determinate the area (*S. aureus* cells) and length (*P. fluorescens* cells), using image analysis (Image-Pro Plus version 6.3 software, 2008 Media Cybernetics Inc., Rockville, MD, USA) by an optical microscope (Olympus CX31, Tokyo, Japan) couple-d to an Infinity 1 camera (Media Cybernetics Inc., Rockville, MD, USA), using a 100× objective [37].

#### 2.10.6. Determination of ROS

ROS were determined according to Dwivedi et al. [38]. Analysis was performed to evaluate the active compounds at two concentrations 90.98 and 1000 μg/mL) based on bacterial growth inhibition (%). A 96-well microplate was filled with bacterial culture (210 μL) at 10^8^ CFU/mL in Mueller-Hinton broth. Then, 90 μL of samples (pure EGCG, Chitosan-P or EGCG-g-chitosan-P) were added, and the plate was incubated at 37 °C for 24 h. After incubation, the medium was removed and the bacterial cells on the bottom of microplate were mixed with 50 μL of DCFH-DA (12.5 μM) and then incubated at 37 °C for 5 min. The ROS generated were determined by fluorescence spectrophotometry (Bio-Rad 3350 microplate reader, Hercules, CA, USA) at an excitation wavelength of 488 nm and an emission wavelength of 535 nm.

### 2.11. In Vitro Determination of the Antioxidant Activity of EGCG-grafted-Chitosan

The same concentrations of EGCG and nanoparticles as those used in the bacterial growth inhibition tests were employed to determine antioxidant activity using ABTS, DPPH, and FRAP assays. The ABTS and DPPH tests were used to evaluate the radical-trapping activity, while the FRAP test was used to evaluate the reducing power of the developed materials [39].

#### 2.11.1. ABTS Radical Scavenging Assay

ABTS radicals were propagated by oxidation of ABTS (7.0 mM) with potassium persulfate (K_2_S_2_O_8_, 4.95 mM) in the dark (12 h) at 25 °C. Then, ABTS was diluted with PBS (0.2 M, pH 7.4) to reach an absorbance value of 0.7 measured at 734 nm. The absorbance value of a mixture of a 20 μL sample and 200 μL of the working solution was measured at 734 nm by a microplate reader (Veloskan™ LUX, Thermo Fisher Scientific, Waltham, MA, USA) after 30 min of reaction. The scavenging activity was reported as ABTS radical inhibition (%) and calculated according to Equation (3).

(3) $$Scavenging\;activity\;\left(\%\right)=\left(1-\left(\frac{Abs_{1}-Abs_{2}}{Abs_{0}}\right)\right)\times 100$$

where the absorbance of water instead of the sample was the control (*Abs*_0_); the absorbance of the sample with ABTS is *Abs*_1_ and the absorbance of the water instead of ABTS is *Abs*_2_. Trolox standard solution was prepared and assayed under the same conditions. Results are expressed in terms of TEAC, which represents the mmol/L Trolox equiv/g sample.

#### 2.11.2. DPPH Radical Scavenging Assay

DPPH radical scavenging assays were performed according to Hu et al. [15]. Pure EGCG or the nanoparticles were homogeneously dispersed in water at the same concentrations as those used to evaluate bacterial growth inhibition. The samples (50 μL) and methanolic DPPH solution (200 μL) at 0.4 mM were added to a 96-well microplate. The reaction was carried out in the dark at 25 °C/30 min. The absorbance was measured at 515 nm by using a microplate reader (Veloskan™ LUX, Thermo Fisher Scientific, Waltham, MA, USA). A Trolox standard solution was prepared and assayed under the same conditions. The scavenging activity was measured as the decrease in absorbance of the DPPH, and it is expressed as percent inhibition of DPPH radicals calculated according to Equation (3). Additionally, antioxidant concentration corresponding to 50% inhibition of the DPPH radical (EC50 DPPH), which was calculated from the graph of scavenging percentage versus concentration of the antioxidant (mg/mL) tested using linear regression.

#### 2.11.3. FRAP Assay

Ferric reducing ability was evaluated based on FRAP assays [40]. The FRAP reagent was prepared from acetate buffer (pH 3.6), 10 mM 2,4,6-tri(2-pyridyl)-s-triazine (TPTZ) solution in 40 mM HCl, and 20 mM iron (III) chloride solution in proportions of 10:1:1 (*v*/*v*/*v*). EGCG or the nanoparticles (20 μL) were added to 280 μL of the FRAP reagent. A Trolox standard solution was prepared and assayed under the same conditions. The absorbance of the reaction mixture was recorded at 638 nm after 30 min using a microplate reader (Veloskan™ LUX, Thermo Fisher Scientific, Waltham, MA, USA). The results are expressed as micromoles of Trolox equivalents per g of dry weight (μmol TE/g).

### 2.12. Statistical Analysis

Data from morphological, particle size, polydispersity, zeta potential, grafting efficiency analysis were expressed as mean ± standard deviation (SD) and subjected to analysis of variance (ANOVA). Statistical analyses of antibacterial and antioxidant activity were carried out using two-way analysis of variance on JMP v.10 for Windows. In both cases, the differences between the means were evaluated by Tukey’s test at the significance level of 5%.

## 3. Results and Discussion

### 3.1. FTIR Analysis

**Figure 1:**
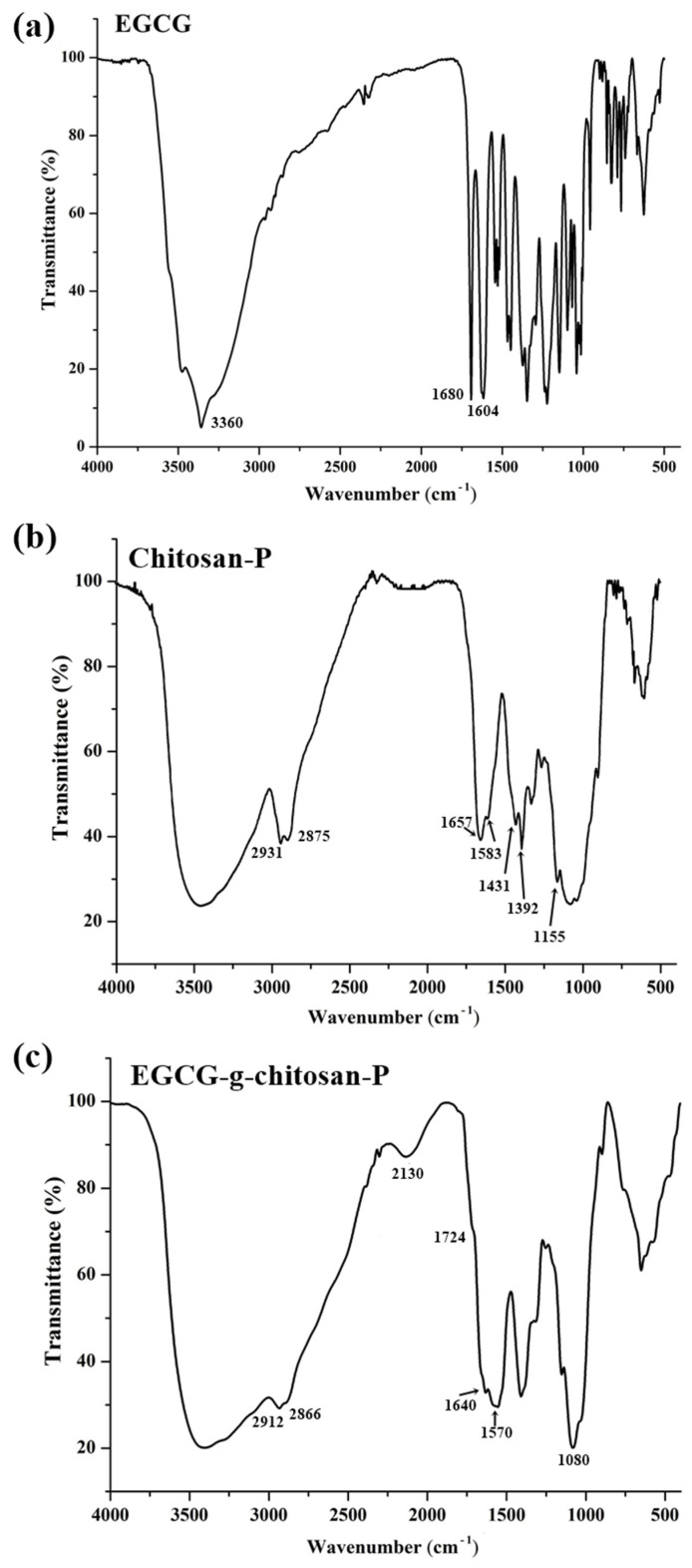
FTIR spectra of epigallocatechin gallate (EGCG) (**a**), chitosan nanoparticles (Chitosan-P) (**b**) and EGCG-grafted-chitosan nanoparticles (EGCG-g-chitosan-P) (**c**).

To verify that the particle synthesis method does not affect the functionalization of chitosan, EGCG-g-chitosan-P was characterized by FTIR spectroscopy. EGCG and Chitosan-P were analyzed as references. Figure 1 and Table 1 show the characteristic bands of EGCG and chitosan. The bands that indicate covalent bonding appear at 2130 cm^−1^ and 1640 cm^−1^ representing imine bond (N=C) stretching vibrations of the Schiff base [41]. Figure 1 shows the above-mentioned bands in the spectrum of the EGCG-grafted-chitosan particles, but not in that of the chitosan particles. In the FTIR spectra of the EGCG-g-chitosan-P (Figure 1), the bands at 1500–1640 cm^−1^ (amide II and amide III groups) are broader than those for the Chitosan-P which could be attributed to the conjugation with the EGCG as well as to the C=C skeletal vibration in the aromatic ring. Additionally, a decrease in band intensity for the primary amine (NH bond) 1550 cm^−1^ and an increase in the band amide I (CO) intensity is observed. Aljawish et al. [35] reported the C=N vibrations (1620 cm^−1^) and C=C stretching vibrations (1640 cm^−1^) characteristics of Schiff base (imine). The Schiff base can be synthesized from an amine and a carbonyl compound to generate an imine. Moreover, EGCG-g-chitosan-P showed bands at 1000–1500 cm^−1^ region indicating the aromatic (C-O) and aliphatic (C-O) bond stretching of EGCG. Moreover, EGCG-g-chitosan-P shows changes in the characteristic peak of saccharide structure at 1080 cm^−1^ (C-O stretching), which indicates that according to Lei et al. [42], hydroxyl radical catalyzed the graft of EGCG onto chitosan chains. These results indicate grafting at the hydroxyl (OH) and primary amine (NH2) groups of chitosan and the phenolic rings of EGGC. These results confirm that the synthesis method does not alter or interfere with the chitosan modification.

### 3.2. Morphological Characterization of Nanoparticles

**Figure 2:**
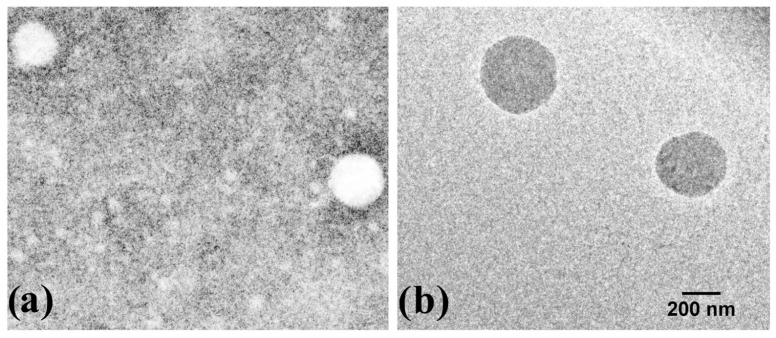
TEM images of (**a**) Chitosan-P and (**b**) EGCG-g-chitosan-P.

Transmission electron microscopy (TEM) is used to determine the size, shape, uniformity, and dispersity of micro and nanomaterials [43]. The micrographs obtained in this study are shown in Figure 2 Chitosan-P (Figure 2a and Figure S1 in Supplementary Material) and EGCG-g-chitosan-P (Figure 2b and Figure S1 in Supplementary Material). The results reveal spherical shapes and smooth surfaces for both nanoparticles. The spherical appearance is due to the nanoprecipitation process, whereby the solvent phase (acetic acid) is displaced by the organic phase (methanol) and the polymer automatically tends to collapse forming nanoparticles or microparticles [23]. The spherical shape and smoother surface of the EGCG-g-chitosan-P could promote their release from polymer matrices, such as films, coatings, or membranes [44].

### 3.3. Particle Size and Polydispersity Index

**Figure 3:**
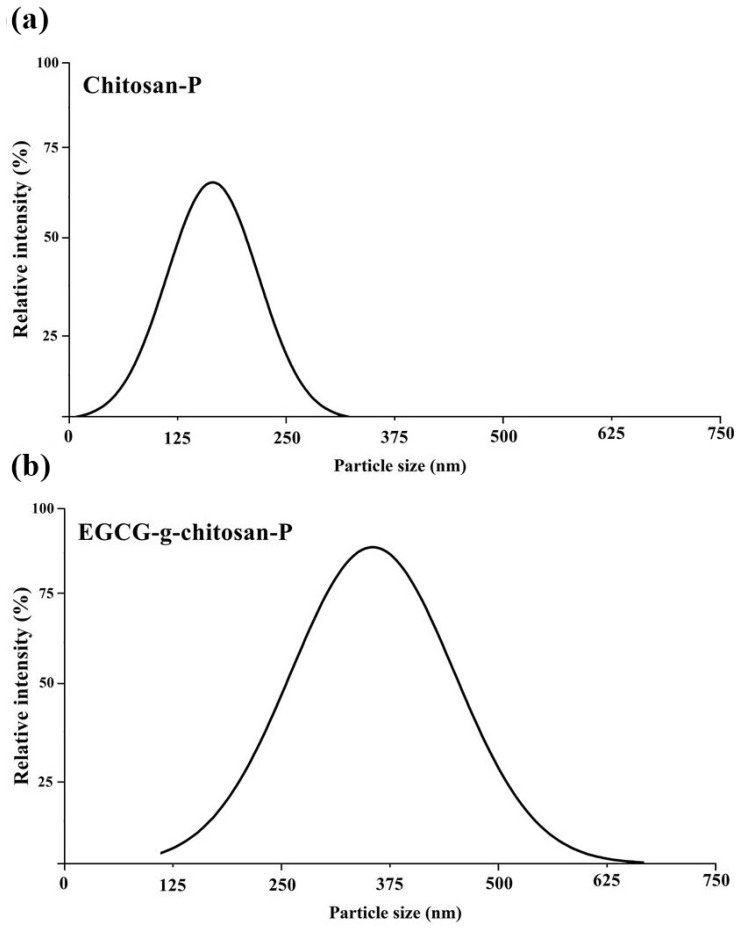
Particle size and polydispersity index results for (**a**) Chitosan-P and (**b**) EGCG-g-chitosan-P.

The results indicate that the Chitosan-P has an average size of 139.10 ± 4.09 nm and a polydispersity index of 0.844 ± 0.130 with a monomodal distribution (Figure 3). These results are in agreement with Barreras-Urbina et al. [23], who reported average size of 50 to 300 nm for nanoparticles synthesized by nanoprecipitation. In the present study, EGCG-g-chitosan-P presents an average size of 362.93 ± 28.36 nm and a polydispersity index of 0.523 ± 0.036 with a monomodal distribution, which indicates a homogenous size (Figure 3). According to Alameh et al. [45], the particle size increases as the molecular weight of the polymer increases. This could be because polymers with high molecular weights exhibit lower diffusion rates in organic phases during particle synthesis. This hypothesis is consistent with previous studies that indicated that the grafting of phenolic compounds to polymers increases their molecular weights [46]. These results are promising, as nanoparticles with sizes <100 nm are considered to be potentially hazardous to biological systems [47].

### 3.4. Zeta Potential (ζ)

The ζ measurement is a key measure of the stability of colloidal dispersions as a result of the degree of electrostatic repulsion between charged particles [48]. Small particles with a high zeta potential will confer stability by resisting aggregation. According to Shegokar et al. [49], ζ values of ≈+30 mV or ≈−30 mV are enough to prevent particle aggregation. In the present study, the ζ value of the Chitosan-P is 53.03 ± 3.76 mV. The surface charge of the nanoparticles decreases slightly (*p* < 0.05) upon EGCG grafting (40.87 ± 0.64 mV). According to Li et al. [50], the decrease in the zeta potential might be ascribed to the possible interaction between catechin with chitosan during the coupling process. This behavior is likely a consequence of the decrease in the number of free amine groups due to the reaction with EGCG to form the conjugate. Therefore, the ζ values for the nanoparticles synthesized in our study show that they exhibit electrostatic repulsion forces that will stabilize them in a dispersing liquid.

### 3.5. EGCG Content in the Nanoparticles

Quantification was performed using the Chitosan-P as control. The control showed no EGCG content. The EGCG content in nanoparticles showed a biological compound loading of 330 ± 6.03 μg EGCG/mg of dry EGCG-g-chitosan-P. The EGCG concentration in the nanoparticles is approximately 100 wt% higher than that achieved in previous studies on EGCG incorporated into chitosan particles [6,7,51].

### 3.6. In Vitro Determination of Antibacterial Activity of Nanoparticles

#### 3.6.1. Bacterial Growth Inhibition (%)

**Figure 4:**
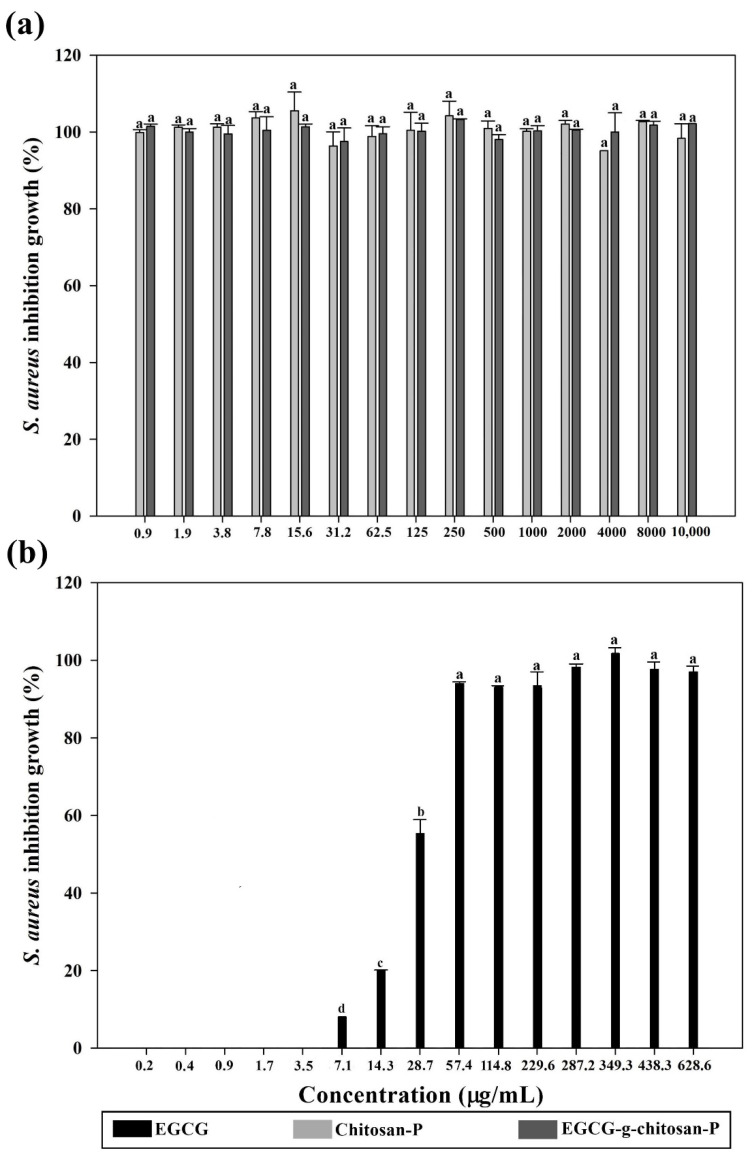
Bacterial growth inhibition (%) of (**a**) nanoparticles (chitosan and EGCG-grafted-chitosan) and (**b**) EGCG pure against *Staphylococcus aureus.* Each value is expressed as the mean ± standard deviation (n = 6). Means with different letters indicate statistically significant differences (*p* < 0.05).

**Figure 5:**
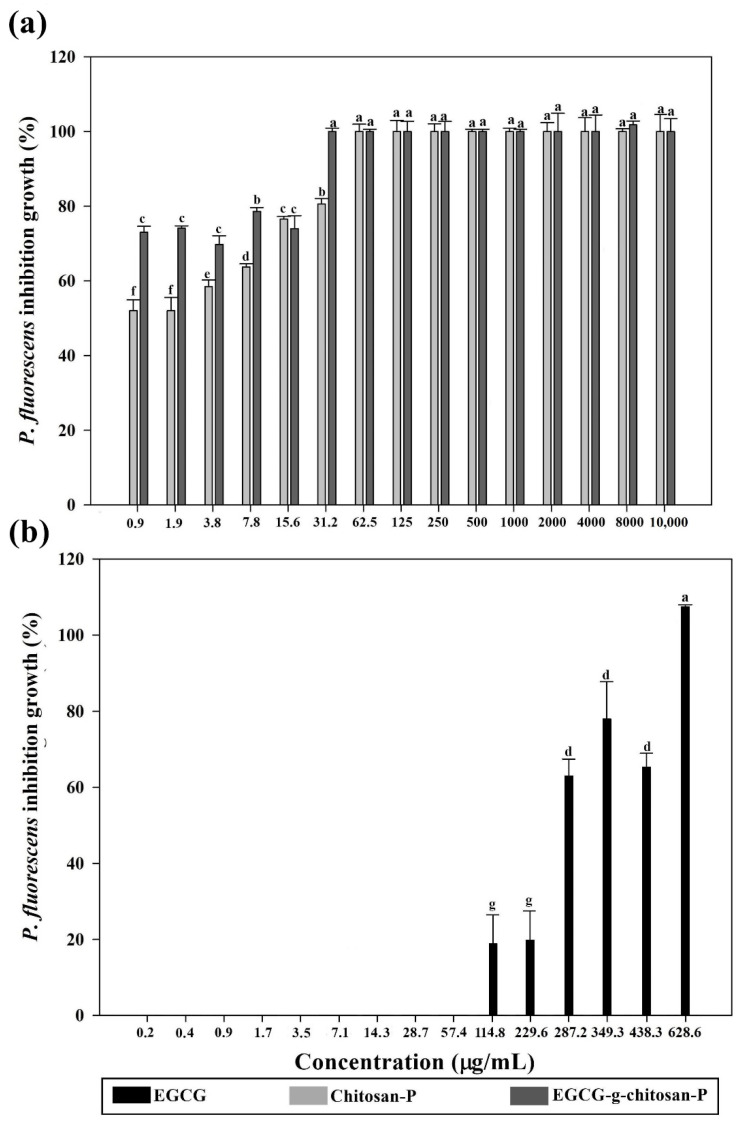
Bacterial growth inhibition (%) of (**a**) nanoparticles (chitosan and EGCG-grafted-chitosan) and (**b**) EGCG pure against *Pseudomonas fluorescens.* Each value is expressed as the mean ± standard deviation (n = 6). Means with different letters indicate statistically significant differences (*p* < 0.05).

Spectrophotometric analysis (Figure 4 and Figure 5) showed that the nanoparticles exhibit higher bacterial growth inhibition than EGCG (0.9 to 2000 μg/mL) against *S. aureus* and *P. fluorescens* (*p* < 0.05) and the effect was concentration-dependent (*p* < 0.05). Chitosan-P and EGCG-g-chitosan-P were effective since 0.98 μg/mL and showed >98% growth inhibition of *S. aureus* (Figure 4). Additionally, EGCG-g-chitosan-P showed higher antibacterial activity (>97% growth inhibition at 31.2 μg/mL) than EGCG (>99% growth inhibition at 628.6 μg/mL) or the Chitosan-P (>99% growth inhibition at 62.5 μg/mL) against *P. fluorescens* (*p* < 0.05) (Figure 5), although the EGCG-g-chitosan Care larger than Chitosan-P, previous studies have reported that a decrease in size particle is an important factor that increases antibacterial activity [32]. This result is important due to the controversy surrounding the size of particles and their toxicological effect [32]. Additionally, the nanoparticles show higher growth inhibition than those of chitosan and bulk EGCG-g-chitosan from 0.98 μg/mL [22]. The results are consistent with those of Liang et al. [26], who reported that bulk chitosan forms structures larger than nano- or microparticles based on chitosan. Thus, the increase in structure size decreases interaction with (and thus internalization into) bacterial cells, which is the principal antibacterial mechanism reported for bulk chitosan.

#### 3.6.2. MICs and MBCs

The MICs and MBCs values for EGCG and the nanoparticles (chitosan and EGCG-grafted-chitosan) against *S. aureus* and *P. fluorescens* are shown in Table 2. EGCG-g-chitosan-P presents lower MIC and MBC values than those of EGCG for both bacterias, and against *Pseudomonas* for Chitosan-P. Moreno-Vasquez et al. [22] reported higher MIC and MBC values for bulk chitosan and EGCG-g-chitosan than those for the particle form, which is due to the increase in the surface area of the nanoparticles compared to that of the bulk material. The EGCG-g-chitosan-P showed a higher antibacterial effect than Chitosan-P synthesized by Chen et al. [32], who reported the antibacterial effect of chitosan nanoparticles (217–235 nm) modified with eugenol and carvacrol (6–40 μg/mL) against *E. coli* and *S. aureus.* Moreover, Saratale et al. [52] reported higher MIC values by silver nanoparticles against *S. aureus* (50 mg/mL) and *E. coli* (40 mg/mL), these results could indicate higher effectiveness by EGCG-g-chitosan-P. The difference in these results could be due to the higher antibacterial activity of pure EGCG than those of pure eugenol and carvacrol. Consequently, the EGCG-grafted-chitosan-P possesses a higher capacity to inhibit bacterial growth, although the nanoparticle size reported by Chen et al. [32] is lower than that in the present work.

The antibacterial activity of the nanoparticles could be due to one of (or a combination of) two factors: (1) particle size, which according to previous studies is a critical factor for antibacterial activity as a decrease in particle size represents an increase in surface area to volume ratio; and (2) the active groups available for interaction with the bacteria cell surface. EGCG-g-chitosan-P possesses free NH_2_ groups and OH groups, which are responsible for the antibacterial activity of chitosan and EGCG, respectively both of them can interact with cell surfaces.

#### 3.6.3. Bacterial Morphometry

**Figure 6:**
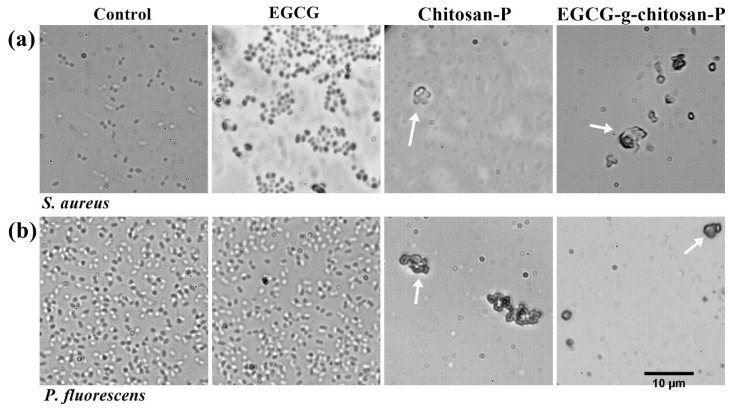
Effect on *S. aureus* (**a**) and *P. fluorescens* (**b**) bacterial morphology by EGCG and nanoparticles at 0.98 μg/mL (magnification = 100×).

Figure 6 shows the effects of a lower concentration (0.98 μg/mL) of the materials against the morphologies of *S. aureus* (see Figure S3 in Supplementary Material) and *P. fluorescens* (see Figure S7 in Supplementary Material). Significant differences (*p* < 0.05) in bacterial morphology are observed upon treatment with the chitosan and EGCG-g-chitosan-P compared to control cells and EGCG (see Figures S4 and S8 in Supplementary Material). *S. aureus* shows a 2.34 ± 0.14 μm^2^ cell area and *P. fluorescens* a 0.92 ± 0.30 μm length cell. However, the cells of both bacteria lose their typical shapes in the presence of Chitosan-P and EGCG-g-chitosan-P. Figure 6 shows cell aggregates, which make it difficult to measure the cell morphology (see Figures S5, S6, S9 and S10 in Supplementary Material), which coincides well with previous studies. For example, Xing et al. [51] used TEM to observe serious damage to the bacterial walls, loss of cytoplasmic material, and changes in bacteria cell shape upon contact with oleoyl-chitosan nanoparticles.

In addition, according to Qi et al. [53], positively charged amine groups interacts with negatively charged bacteria cell membranes, leading to the leakage of bacterial constituents (i.e., peptidoglycan and proteins) causing cell agglutination. Furthermore, a previous study indicated that the OH groups of EGCG interact with proteins and/or enzymes in the cell walls and interfere with the synthesis of cell walls and membranes [4].

#### 3.6.4. Reactive Oxygen Species (ROS)

**Figure 7:**
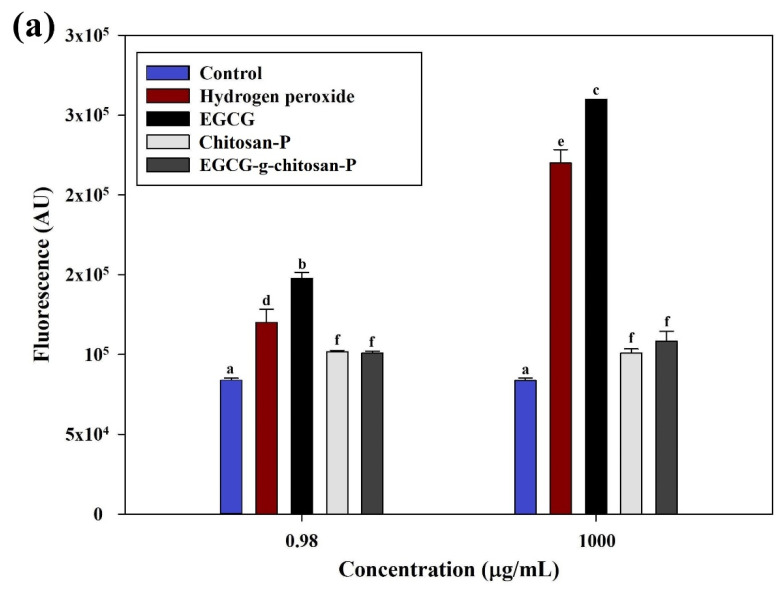

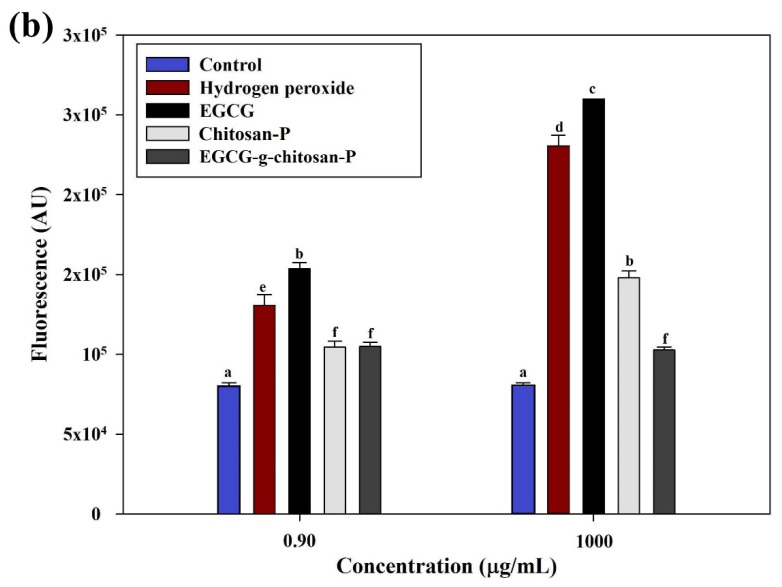
Measurement of ROS produced by *S. aureus* (**a**) and *P. fluorescens* (**b**). Each value is expressed as the mean ± standard deviation (n = 4). Means with different letters indicate statistically significant differences (*p* < 0.05).

The toxicity of nano- and micromaterials is frequently attributed to ROS and ROS-induced damage [54]. ROS can be determined with the oxidation-sensitive fluorescence probe 2,7-dichlorofluoresce indiacetate (DCFH-DA). The DCFH-DA passively diffuses through the cell membrane into the cell and forms non-fluorescent 2,7-dichlorohydrofluorescein (DCFH). The DCFH reacts with ROS to form the fluorescent product 2,7-dichlorofluorescein (DCF), which is trapped inside the cell, making it fluorescent [38]. Figure 7 shows the results of ROS measurement for *S. aureus* and *P. fluorescens* treated with different compounds at 0.98 and 1000 μg/mL. The *S. aureus* cells show an increase (*p* < 0.05) in ROS measurement, as well as an increase in EGCG and hydrogen peroxide concentration. However, an increase in particle concentration shows no additional effect (*p* > 0.05). This could be due to the viability of cells treated with nanoparticles at 1000 μg/mL being too low to produce a significant increase in fluorescence intensity.

The results are consistent with bacterial growth inhibition higher than 95% at 0.98 μg/mL. Previous studies have reported that ROS and ROS-induced damage are the major factors in the inhibition of bacteria. ROSs are produced when nanoparticles interact with bacterial culture [55,56]. Bacterial cells in contact with nanoparticles exhibit inhibition of respiratory enzyme expression, which promotes the generation of ROSs and consequently damages the cell. The ROS produced can irreversibly damage bacteria components, such as cell walls, membranes, and DNA, resulting in bacterial death. Chen et al. [32], reported a similar effect for copper/titanium dioxide nanoparticles at higher concentrations (21.4 μg/mL) against *Escherichia coli* and *Staphylococcus aureus.* These results indicate that the nanoparticles synthesized in the present study exhibit antibacterial activities comparable to that of metal oxide nanoparticles. Furthermore, according to our knowledge, there are no studies in the literature on the effects of nanoparticles synthesized from chitosan-grafted-phenolic compounds on the ROSs produced by bacterial cultures involved in food spoilage and food-borne pathogens.

### 3.7. In Vitro Determination of the Antioxidant Activity of EGCG-g-Chitosan-P

Antioxidants present their action through various mechanisms, such as the scavenging of metals, trapping of free radicals, inactivators of peroxides, the reduction of compounds, quencher of secondary oxidation products, among others [57]. Multiple methods have been developed for its evaluation, however, this variety of mechanisms, and other related factors such as its chemical structure and possible interactions, have made it difficult to apply a single method for its evaluation [58]. Due to this, the evaluation of antioxidant activity requires considering several methods that differ in their reaction mechanism. In this study, the antioxidant activity was evaluated by the ABTS and DPPH assays for its inactivation mechanism, which involves the hydrogen atom transfer reaction. The FRAP test was used to evaluate the reducing power of the antioxidant materials developed.

#### 3.7.1. ABTS Radical Scavenging Assay

**Figure 8:**
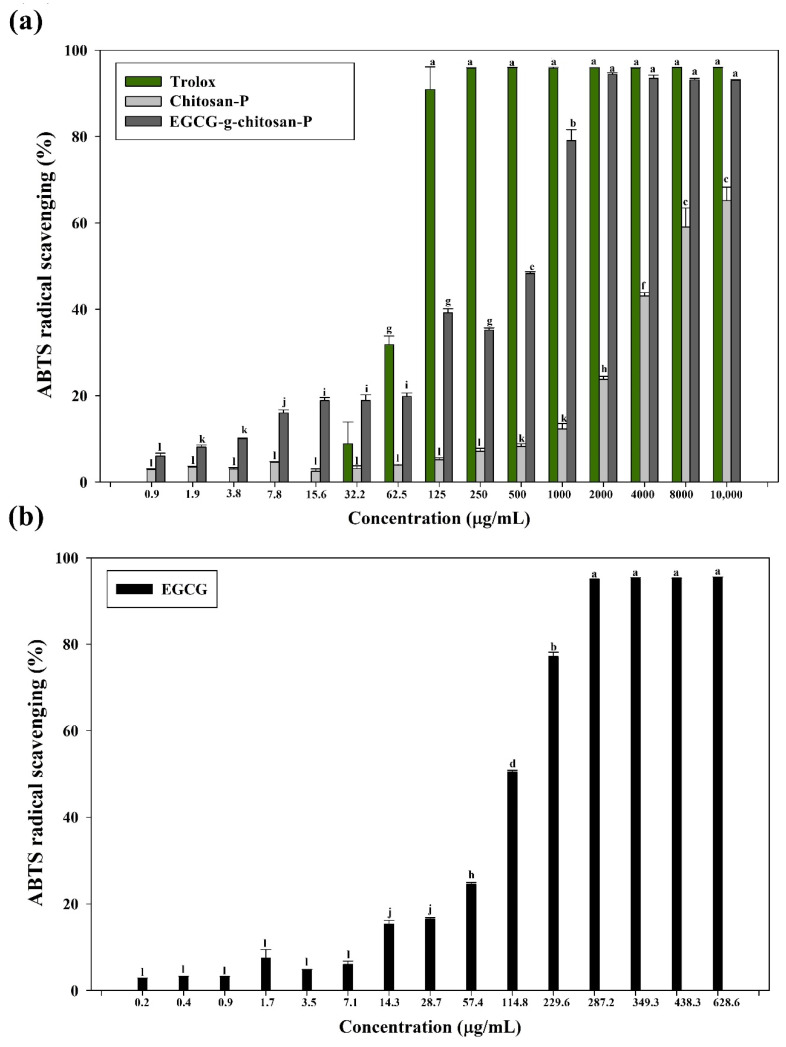
Scavenging ability of (**a**) Trolox and nanoparticles (chitosan and EGCG-grafted-chitosan) and (**b**) EGCG pure on ABTS radicals. Each value is expressed as the mean ± standard deviation (n = 4). Means with different letters indicate statistically significant differences (*p* < 0.05).

This assay is based on the discoloration of a green ABTS radical solution. The discoloration degree depends on the electron-donating ability of the nanoparticles. The results, which are shown in Figure 8, indicate that Chitosan-P exhibits a 63% inhibition at the highest concentration tested (10,000 μg/mL).

However, the activity of EGCG-g-chitosan-P is dependent on concentration (*p* < 0.05), causing 94.3% inhibition of ABTS radicals at 2000 μg/mL, which indicates the ability of chitosan modified to donate protons. EGCG-g-chitosan-P exhibit higher inhibition of ABTS radicals compared to materials developed in previous studies, such as Aljawish et al. [35], who reported that the ABTS radical scavenging activity of chitosan functionalized with ferulic acid (25 μg/mg of chitosan) and ethyl ferulate (17 μg/mg of chitosan) is 51.2 ± 1.5%. It is worthy of note that the EGCG-grafted-chitosan-P in the present study has less active compound grafted to the chitosan than that in Aljawish et al. [35]. Additionally, Table 3 shows the antioxidant activities of EGCG and nanoparticles based on chitosan and EGCG grafted-chitosan in terms of TEAC, which represents the mmol/L Trolox equiv/g sample, a notable increase in the antioxidant activity of the Chitosan-P is observed in comparison to EGCG and EGCG-grafted-chitosan-P. A similar value was reported by Dulong et al. [59], who evaluated the effect of carboxymethylpullulan grafted with ferulic acid.

#### 3.7.2. DPPH Radical Scavenging Assay

**Figure 9:**
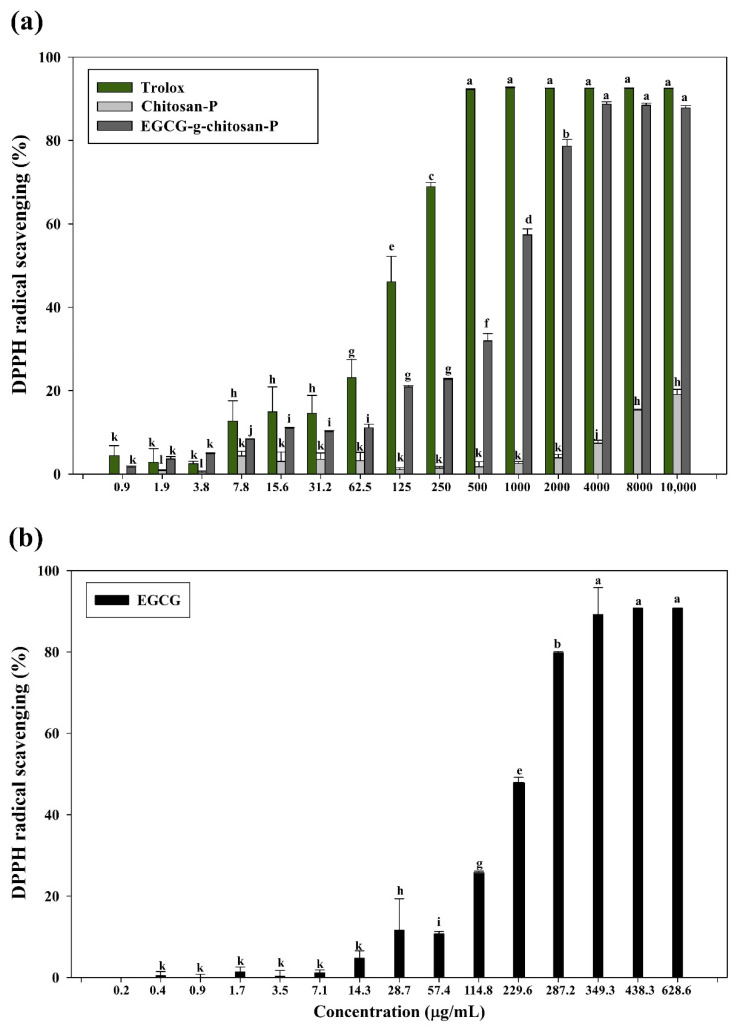
Scavenging ability of (**a**) Troloxand nanoparticles (chitosan and EGCG-grafted-chitosan) and (**b**) EGCG pure. Means with different letters indicate statistically significant differences (*p* < 0.05).

This analysis is based on the discoloration of a yellow DPPH radical solution upon the formation of diphenylpicrylhydrazine. The degree of discoloration will depend on the hydrogen-donating ability of the antioxidant molecule. The DPPH inhibition capacities of pure EGCG and the nanoparticles are shown in Figure 9 and Table 3. The results indicate that the Chitosan-P exhibits lower inhibition activity against DPPH radicals. Conversely, the EGCG-grafted-chitosan-P showed a concentration-dependent effect (*p* < 0.05), which could be attributed to the EGCG grafted to the chitosan backbone. However, the DPPH inhibition exhibited by EGCG-grafted-chitosan-P is low compared to that of pure EGCG, probably due to the hydroxyl groups in the EGCG, which are responsible for the antioxidant activity, being involved in its bonding to chitosan. Similar behavior has been reported for methacrylic acid functionalized with ferulic acid by DPPH radical inhibition [60]. The above behavior could also be due to the difference in the mobility of free radicals when EGCG is covalently linked to chitosan (i.e., the effect of steric hindrance around the EGCG molecules and/or higher viscosity) and the exposure of the free radicals to EGCG, as described previously [35,56]. Table 3 shows the results expressed as the antioxidant concentration corresponding to 50% inhibition of DPPH radicals (mg/mL). The EC_50_ value for EGCG-grafted-chitosan-P is two-fold lower than those reported by Chen et al. [32], who evaluated the antioxidant activity of carvacrol-grafted chitosan nanoparticles (EC_50_ = 2.6 mg/mL). Additionally, Aljawish et al. [35] reported the scavenging activity of ferulic acid-chitosan derivative that is poorer than that for EGCG-g-chitosan-P (1.50 ± 0.04 mg/mL). The above behavior could be attributed to the superior antioxidant properties of EGCG compared to those of ferulic acid and carvacrol.

#### 3.7.3. Ferric-Reducing Antioxidant Power (FRAP) Assay

The FRAP assay is based on an active compound’s capacity to directly reduce Fe(III) to Fe(II). Table 2 shows the values obtained for EGCG, Chitosan-P, and EGCG-g-chitosan-P, which indicate that the increase (*p* < 0.05) in the capacity of EGCG-grafted-chitosan-P to directly reduce Fe(III) could be due to the EGCG. Previous studies have demonstrated that EGCG is an effective iron chelator [61]. Furthermore, previous studies have shown that more than one method is required to calculate the antioxidant capacity of active compounds in vitro, because of the differences in their ability to produce free radicals [62]. According to the antioxidant activity results, the mechanisms by which EGCG-g-chitosan particles exert their activity involve at least hydrogen atom transfer and reducing power, as described by Boulmokh et al. [63] for EGCG. Recently, it has been reported that the high antioxidant activity of EGCG is due to the gallate moiety and 4’-OH in the B ring present in its structure [63], which are related to the ability to quench free radicals by developing resonance-stabilized phenoxyl moieties [61].

## 4. Conclusions

Spherical and smooth-surfaced nanoparticles based on chitosan modified by EGCG were obtained. Their zeta potential indicated their chemical stability in colloidal systems. These results show that the use of the nanoprecipitation technique to synthesize nanoparticles made of chitosan covalently grafted to EGCG is a successful and environmentally safe method for improving the antibacterial and antioxidant activities of EGCG and chitosan. These results reveal that EGCG-g-chitosan-P exhibited greater antibacterial activity against *Pseudomonas* than Chitosan-P and EGCG. Evidence of ROS production by bacterial cells in contact with the different treatments evaluated was found, which may be related to damage of their cellular components, resulting in bacterial growth inhibition. Furthermore, the results show a higher antioxidant activity of EGCG-g-chitosan-P than Chitosan-P. It was found that the modified chitosan nanoparticles present antioxidant activity through hydrogen atom transfer mechanism and reducing power. Finally, it was observed that the antibacterial activity of the covalently grafted EGCG-g-chitosan nanoparticles is mainly due to chitosan, while their antioxidant activity is mainly due to EGCG. Taking into account the increasing demand for effective and natural antioxidants and antibacterial, this study demonstrated that EGCG-g-chitosan nanoparticulated systems have potential applications as functional materials for biomedical, food packaging, and food preservation purposes, as well as novel nutraceuticals and pharmaceutical compounds with antioxidant activity.

## Figures and Tables

**Table 1:** Peaks position and assignment of main IR absorption bands of EGCG and nanoparticles of chitosan and EGCG-grafted-chitosan.

Sample	Peak Position Wavenumber (cm^−1^)	Peak Assignment
EGCG	3650–3200	OH group (aromatic ring)
	1600–1450	C=C group (aromatic ring)
	1148	C=O group (heterocyclic pyranose)
Chitosan-P	3600–3200	OH group
	2931–2875	CH group
	1657	C=O (amide I group)
	1583	N-H (amide II group)
	1431	C-N (amide III group)
	1400–1200	C–C–H, O–C–H, and C–O–H bending vibrations mode of mono or polysaccharides
	1156–1030	C-O-C (glycosidic bond)
	1080	C-O (saccharide structure)
EGCG-g-chitosan-P	3650–3200	OH group
	2912–2866	CH group
	2130	C=N (imine bond)
	1640	C=N (imine bond)
	1600–1500	C=C group (aromatic ring)
	1570	N-H (amide II group)
	1416	C-N (amide III group)
	1080	C-O (saccharide structure)
	1060	C-O- group

**Table 2:** Minimal inhibitory concentration (MIC) and minimal bactericide concentration (MBC) of EGCG and nanoparticles (chitosan and EGCG-grafted-chitosan) against the tested bacteria.

Antibacterial Activity	Bacteria	Concentration (μg/mL)		
		EGCG	Chitosan-P	EGCG-g-Chitosan-P
**Minimal Inhibitory Concentration (MIC)**	*S. aureus*	62.5 ± 0.21 ^a^	<0.98 ^b^	<0.98 ^b^
	*P. fluorescens*	500 ± 0.01 ^a^	31.2 ± 0.3 ^b^	15.6 ± 0.9 ^c^
**Minimal Bactericide Concentration (MBC)**	*S. aureus*	125 ± 0.00 ^a^	<0.98 ^b^	<0.98 ^b^
	*P. fluorescens*	1000 ± 0.01 ^a^	62.5 ± 0.51 ^b^	31.2 ± 0.9 ^c^

Note: the data are represented as mean values ± standard deviation (n = 6). Different letters within the same row indicate statistically significant differences (*p* < 0.05).

**Table 3:** Antioxidant activity of EGCG and nanoparticles based on chitosan and EGCG-grafted-chitosan.

Samples	TEAC (mmol/L Trolox equiv/g Sample)	DPPH (EC_50_ mg/mL)	FRAP (μmol TE/g)
EGCG	0.39 ± 0.01 ^a^	1.16 ± 0.01 ^a^	261.61 ± 14.81 ^a^
Chitosan-P	0.03 ± 0.01 ^b^	>10 ^b^	39.56 ± 5.26 ^b^
EGCG-g-chitosan-P	0.40 ± 0.03 ^a^	1.00 ± 0.01 ^c^	310.75 ± 15.96 ^c^

Note: the data are represented as mean values ± standard deviation (n = 4). Different letters within the same row indicate statistically significant differences (*p* < 0.05).

## Data Availability

The data presented in this study are available on request from the corresponding author.

## Supplementary Materials

The following are available online at https://www.mdpi.com/article/10.3390/polym13091375/s1, Figure S1: TEM images of chitosan-P; Figure S2: TEM image of EGCG-g-chitosan-P; Figure S3. Effect on *S. aureus* control (magnification = 100×); Figure S4. Effect on *S. aureus* bacterial morphology by EGCG at 0.9 mg/mL (magnification = 100×); Figure S5. Effect on *S. aureus* bacterial morphology by Chitosan-P at 0.9 mg/mL (magnification = 100×); Figure S6. Effect on *S. aureus* bacterial morphology by EGCG-g-chitosan-P at 0.9 mg/mL (magnification = 100×); Figure S7. Effect on *P. fluorescens* control (magnification = 100×); Figure S8. Effect on *P. fluorescens* bacterial morphology by EGCG at 0.9 mg/mL (magnification = 100×); Figure S9. Effect on *P. fluorescens* bacterial morphology by Chitosan-P at 0.9 mg/mL (magnification = 100×); Figure S10. Effect on *P. fluorescens* bacterial morphology by EGCG-g-chitosan-P at 0.9 mg/mL (magnification = 100×).
