# Supplementary material for: Characterization of Epigallocatechin-Gallate-Grafted Chitosan Nanoparticles and Evaluation of Their Antibacterial and Antioxidant Potential
Source: Polymers (Basel). 2021 Apr 23;13(9):1375. doi: 10.3390/polym13091375 (PMC8122830; doi:10.3390/polym13091375)
Supplement: Supplementary file 1 [file polymers-13-01375-s001.zip › polymers-1172484-supplementary.pdf]

Supplementary Material

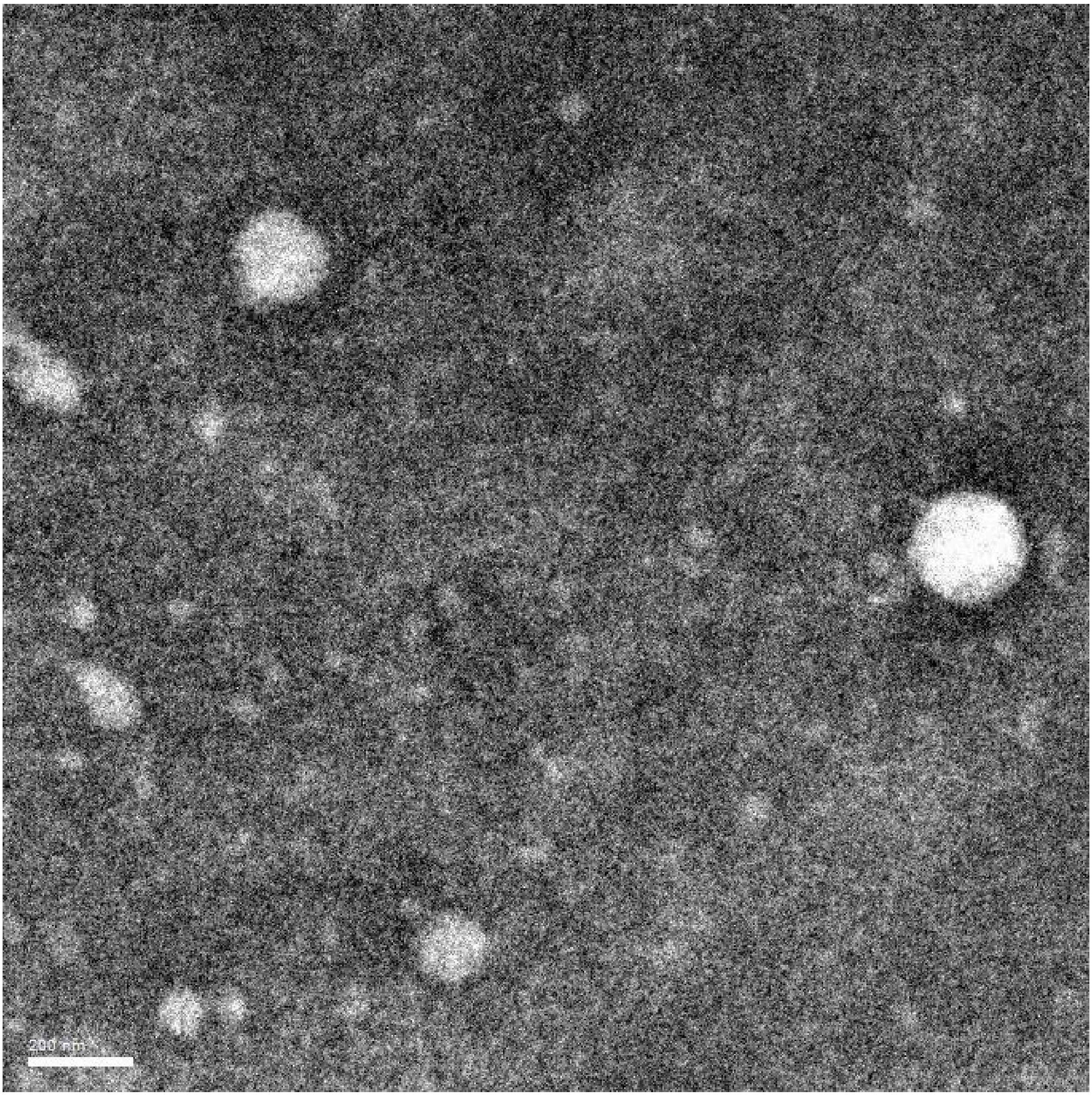

Figure S1. TEM images of Chitosan-P.

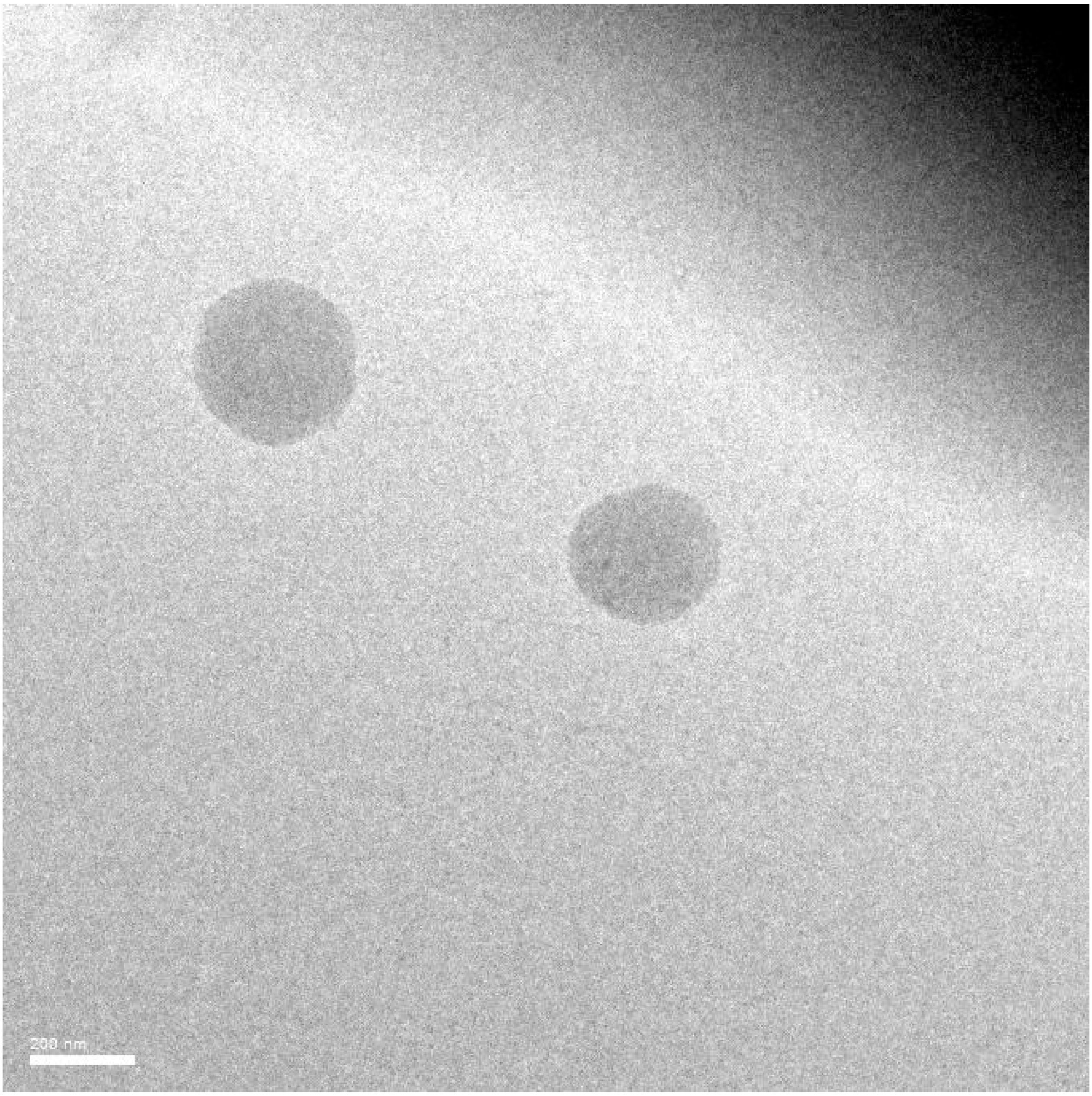

Figure S2. TEM image of EGCG-g-chitosan-P.

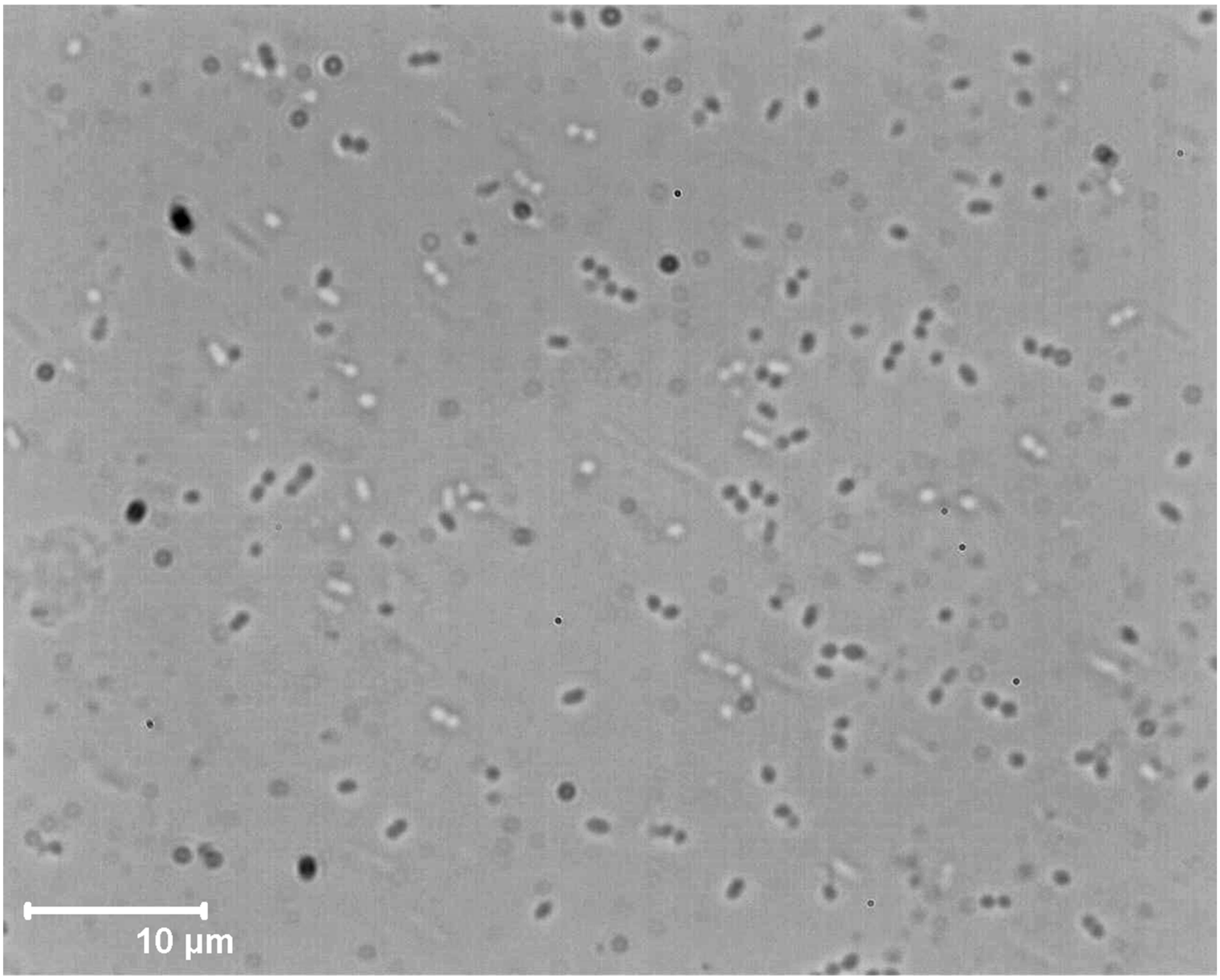

Figure S3. Morphology of *S. aureus* (control) (magnification =100x).

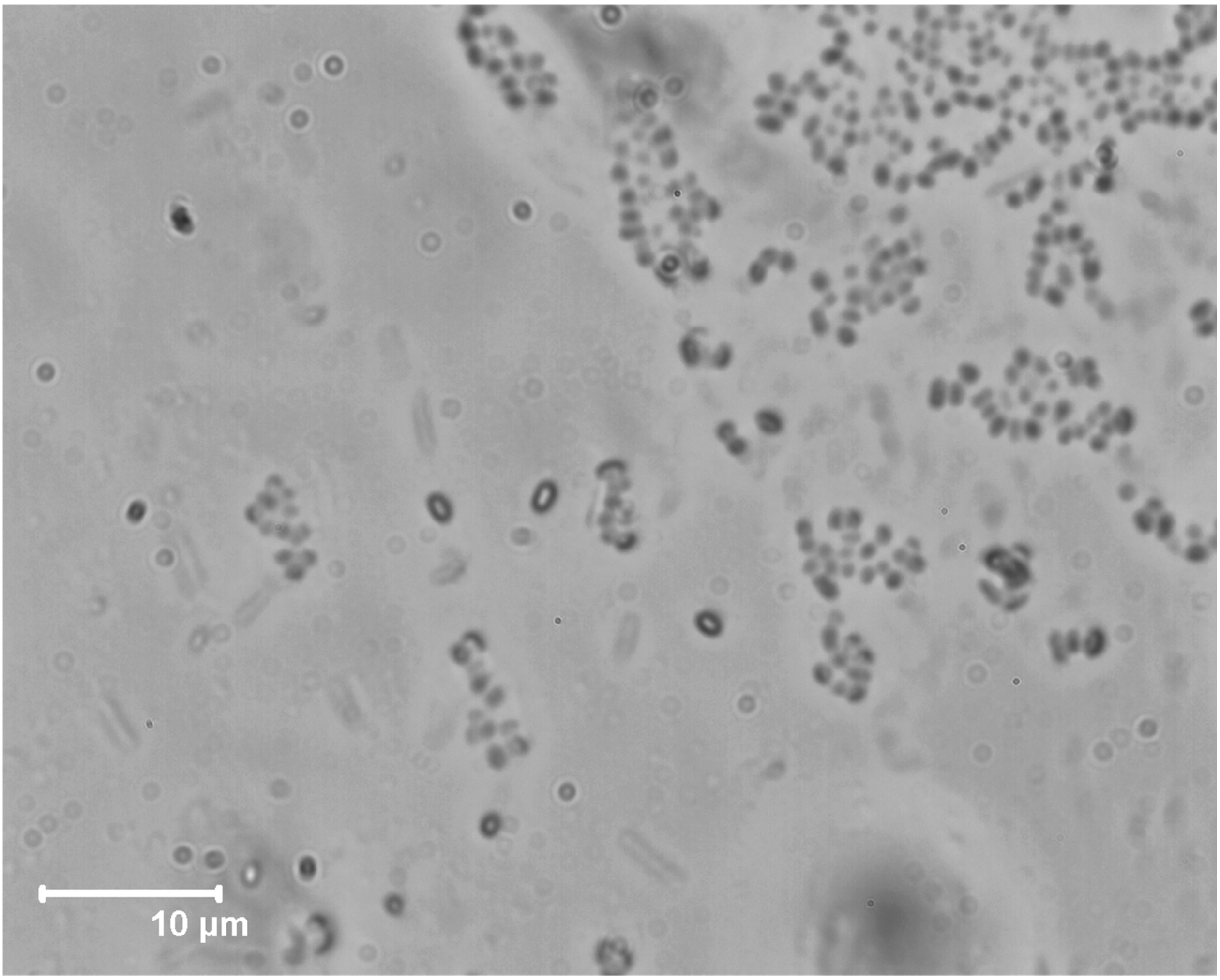

Figure S4. Effect of EGCG at 0.9 µg/mL on *S. aureus* bacterial morphology (magnification =100x).

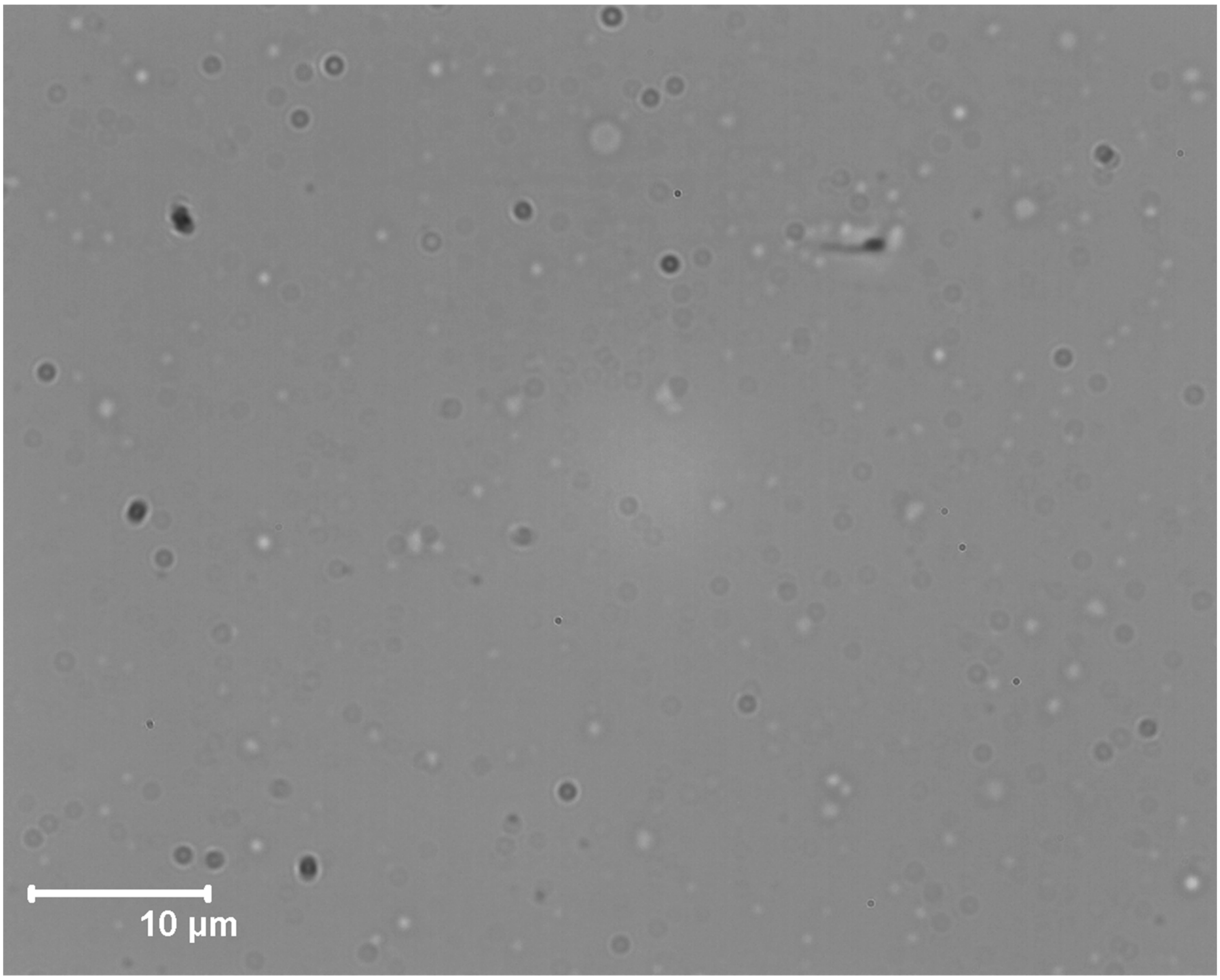

Figure S5. Effect of Chitosan-P at 0.9 µg/mL on *S. aureus* bacterial morphology (magnification =100x).

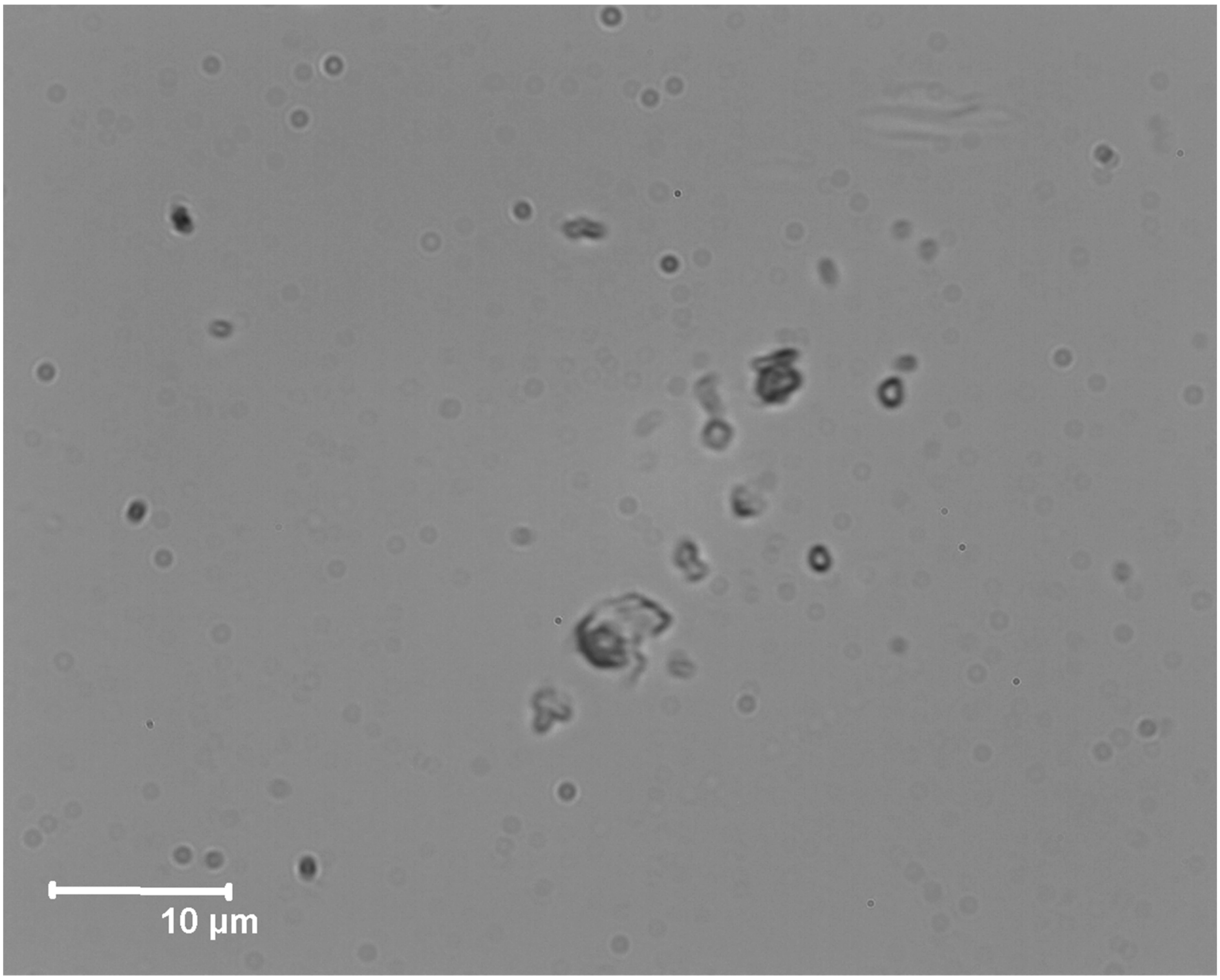

Figure S6. Effect of EGCG-g-chitosan-P at 0.9 µg/mL on *S. aureus* bacterial morphology (magnification =100x).

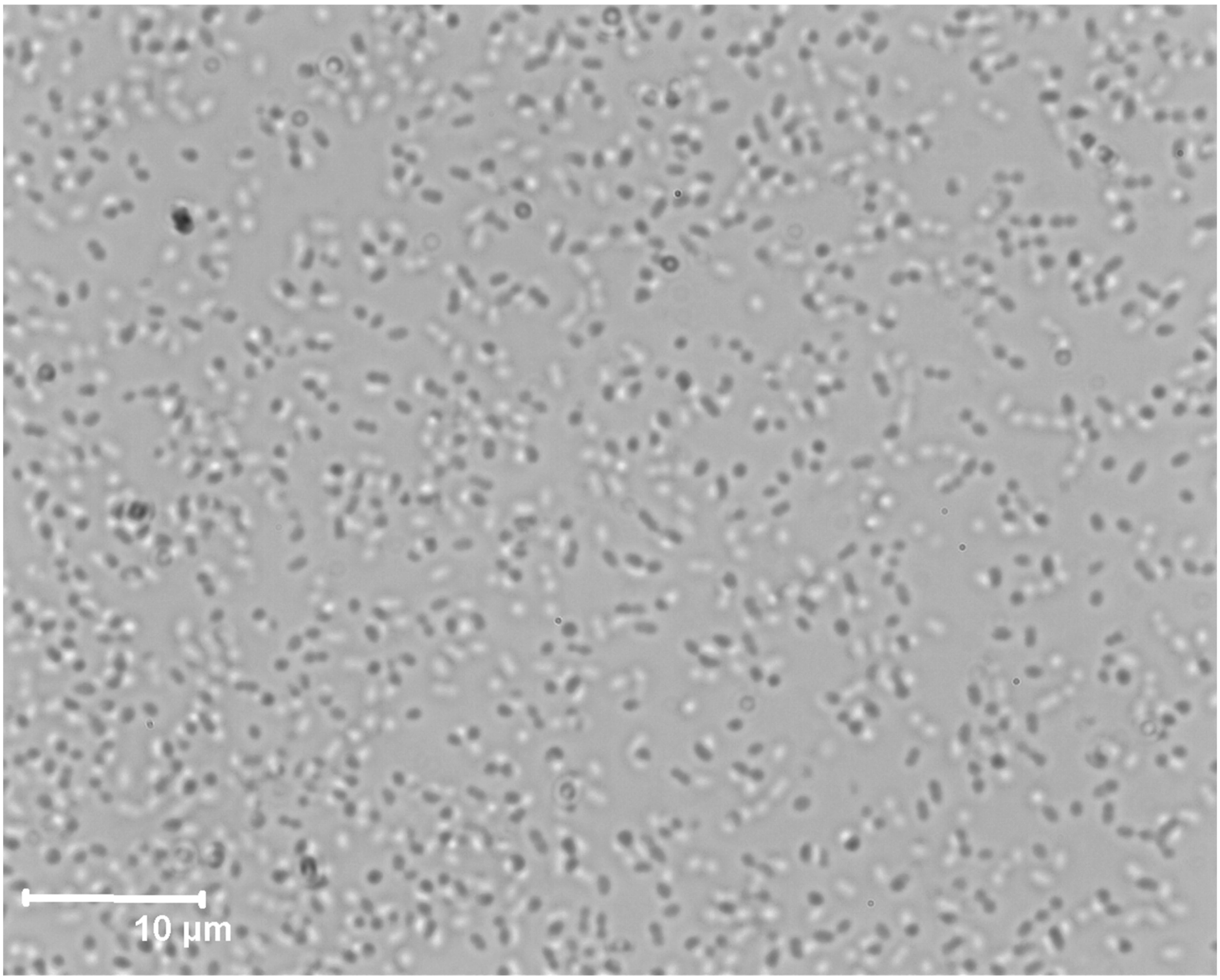

Figure S7. Morphology of *P. fluorescens* (control) (magnification =100x).

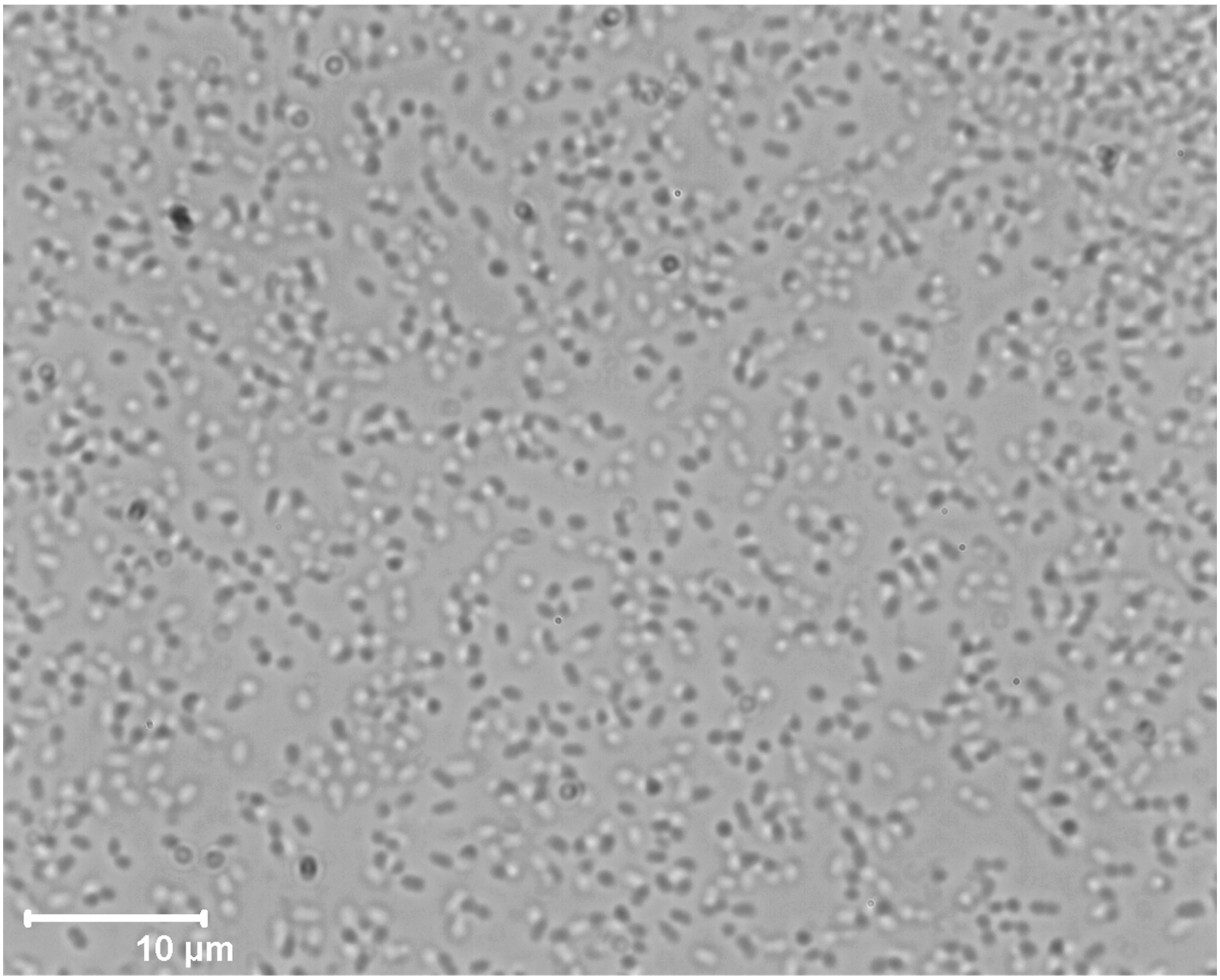

Figure S8. Effect of EGCG at 0.9 µg/mL on *P. fluorescens* bacterial morphology (magnification =100x).

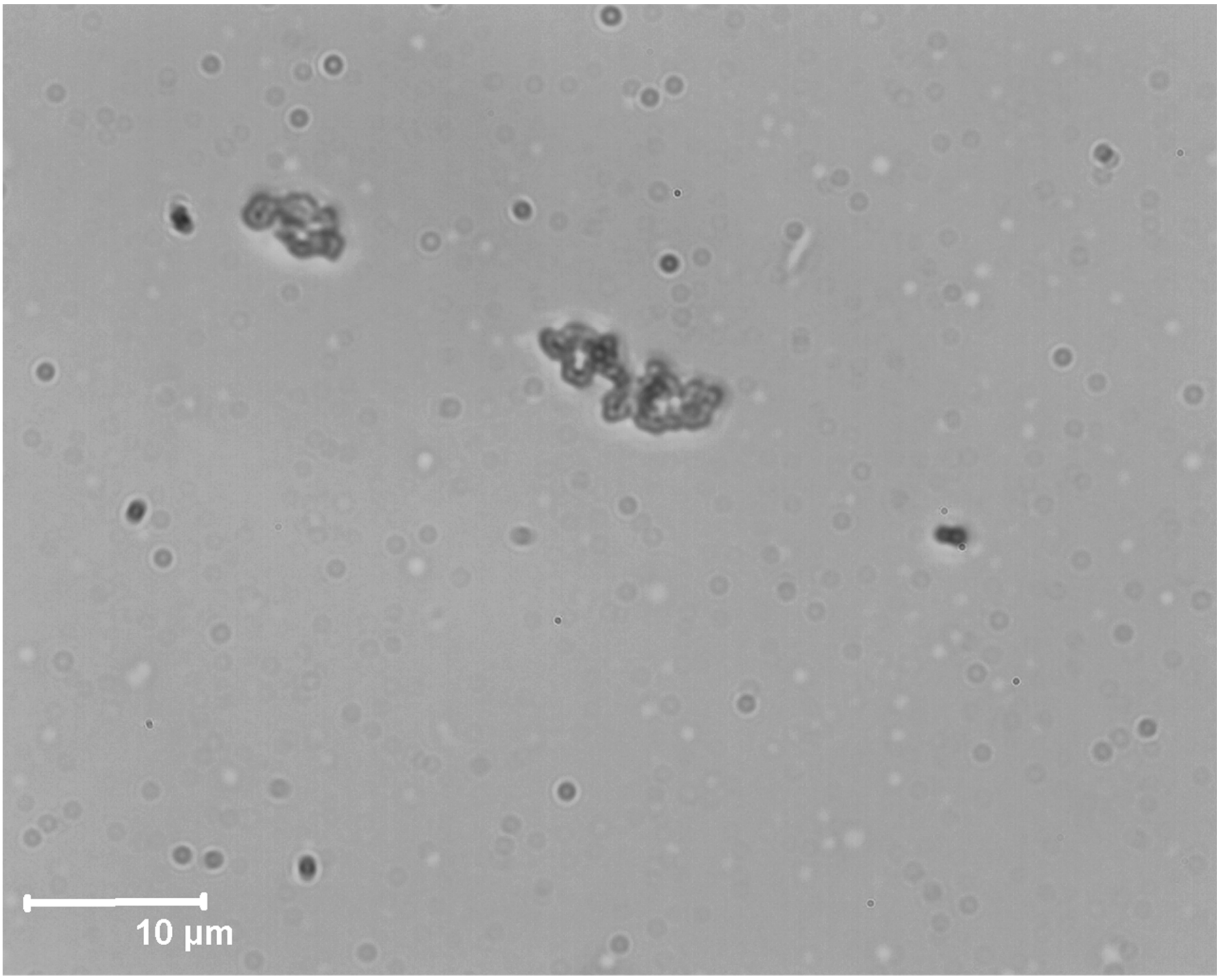

Figure S9. Effect of Chitosan-P at 0.9 µg/mL on *P. fluorescens* bacterial morphology (magnification =100x).

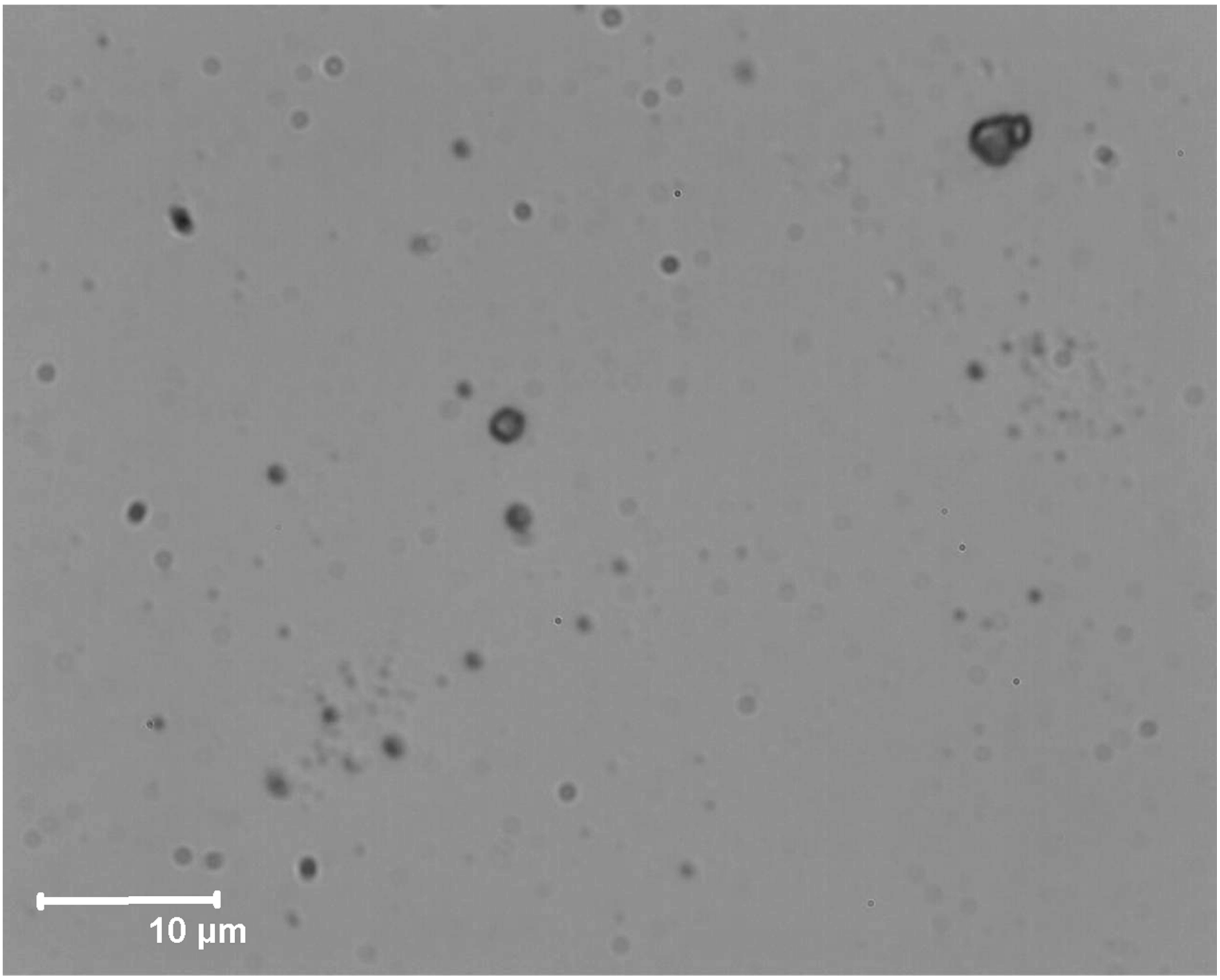

Figure S10. Effect of EGCG-g-chitosan-P at 0.9 µg/mL on *P. fluorescens* bacterial morphology (magnification =100x).
